# IL-36 receptor agonist and antagonist imbalance drives neutrophilic inflammation in COPD

**DOI:** 10.1172/jci.insight.155581

**Published:** 2022-08-08

**Authors:** Jonathan R. Baker, Peter S. Fenwick, Carolin K. Koss, Harriet B. Owles, Sarah L. Elkin, Jay Fine, Matthew Thomas, Karim C. El Kasmi, Peter J. Barnes, Louise E. Donnelly

**Affiliations:** 1 National Heart and Lung Institute, Imperial College London, London, United Kingdom.; 2 Boehringer Ingelheim Pharma GmbH & Co. KG, Biberach, Germany.; 3 Department of Respiratory Medicine, Imperial College Healthcare Trust, London, United Kingdom.; 4 Boehringer Ingelheim Pharmaceuticals Inc., Ridgefield, Connecticut, USA.

**Keywords:** Cell Biology, Pulmonology, COPD, Cellular immune response, Cytokines

## Abstract

Current treatments fail to modify the underlying pathophysiology and disease progression of chronic obstructive pulmonary disease (COPD), necessitating alternative therapies. Here, we show that COPD subjects have increased IL-36γ and decreased IL-36 receptor antagonist (IL-36Ra) in bronchoalveolar and nasal fluid compared with control subjects. IL-36γ is derived from small airway epithelial cells (SAEC) and is further induced by a viral mimetic, whereas IL-36Ra is derived from macrophages. IL-36γ stimulates release of the neutrophil chemoattractants CXCL1 and CXCL8, as well as elastolytic matrix metalloproteinases (MMPs) from small airway fibroblasts (SAF). Proteases released from COPD neutrophils cleave and activate IL-36γ, thereby perpetuating IL-36 inflammation. Transfer of culture media from SAEC to SAF stimulated release of CXCL1, which was inhibited by exogenous IL-36Ra. The use of a therapeutic antibody that inhibits binding to the IL-36R attenuated IL-36γ–driven inflammation and cellular crosstalk. We have demonstrated a mechanism for the amplification and propagation of neutrophilic inflammation in COPD and have shown that blocking this cytokine family via a IL-36R neutralizing antibody could be a promising therapeutic strategy in the treatment of COPD.

## Introduction

Chronic obstructive pulmonary disease (COPD) is a major global health burden that affects ~10% of people older than 45 years and, prior to the current SARS-CoV-2 pandemic, is the third most common cause of death in the world (1). COPD is a chronic inflammatory lung disease, associated with increased numbers of inflammatory cells, including macrophages, neutrophils, and lymphocytes (2). Inflammation occurs predominantly in the lung parenchyma and peripheral airways, and it results in irreversible and progressive airflow limitation due to small airway fibrosis and parenchymal destruction (emphysema). Current therapies targeting the inflammatory mediators of COPD have been poorly effective clinically and do not reduce disease progression, so alternative antiinflammatory approaches are greatly needed (3).

IL-36 cytokines belong to the IL-1 superfamily and comprise 3 receptor agonists (IL-36α, IL-36β, and IL-36γ) and 2 receptor antagonists (IL-36Ra and IL-38). The IL-36 receptor comprises a heterodimer of IL-36R and the IL-1 receptor accessory protein (IL-1RAcP), so that IL-36R competes with the IL-1R for IL-1RAcP (4–6). Binding of IL-36 agonists to the receptor leads to the recruitment of IL-1RAcP and subsequent downstream signaling via NF-κB and mitogen-activated protein kinase (MAPK) pathways (7). IL-36 cytokines may, therefore, play an important role in the chronic release of proinflammatory cytokines and chemokines in inflammatory conditions, such as COPD.

IL-36α, IL-36β, IL-36γ, and IL-36Ra are all secreted in an inactive form and require N-terminal cleavage by serine proteases to induce a 500-fold increase in their activity (8). IL-36 isoforms are cleaved by neutrophil products — specifically neutrophil elastase, proteinase-3, and cathepsin G — with each exhibiting selectivity (9). Neutrophils are markedly increased in the lungs and airways of subjects with COPD and are associated with increased secretion of several proteases (10), which are further increased during acute exacerbations (2, 11). In COPD, this could lead to persistent activation of IL-36 and subsequent downstream inflammatory signaling.

Elevated levels of IL-36 cytokines have been reported in COPD, with IL-36α and IL-36γ being elevated in plasma and bronchoalveolar lavage fluid (BALF) from smokers with and without COPD compared with healthy controls, in a small population of only 5 subjects with mild disease (12). IL-36γ is also increased in sputum samples from COPD subjects with a neutrophilic phenotype (13). A similar association of IL-36γ with neutrophilia has been reported in subjects with obstructive lung disease, whereas a decrease in IL-36γ is associated with eosinophilia (14). Along with the importance of examining the levels of receptor agonists, a recent study suggests that there is a reduction in IL-36Ra in both the serum and the sputum of pediatric asthmatic subjects (15). These authors also show that intranasal administration of IL-36Ra in a murine model of asthma reduced airway hyperresponsiveness and inflammatory cell infiltrates into the lung (15).

Most of the current understanding of the role of IL-36 in respiratory disease has come from in vivo mouse models of lung disease. Intratracheal instillation of *Pseudomonas aeruginosa* in mice increased expression of IL-36α and IL-36γ in BALF and lung homogenates (16). Subsequent in vitro experiments confirmed that this was due to increased expression by alveolar macrophages and epithelial cells (17). Of note, KO of either *IL-36r* or *IL-36g*, but not *IL-36a*, protected against lung damage and increased survival time caused by infection of this bacterium (17). By contrast, when mice were exposed to an intranasal influenza viral challenge, there was induction of IL-36α but not IL-36γ protein in the lung, although *IL-36g* mRNA was induced (16). Others have suggested that IL-36γ is upregulated following viral infection and is a protective mechanism due to skewing of macrophage phenotypes toward a more proresolving M2 phenotype (18). We have recently shown that intratracheal delivery of IL-36γ into the lung of mice increases neutrophil chemokines and numbers. Using a COPD exacerbation model of cigarette smoke exposure in combination with influenza H1N1, we showed that IL-36R–deficient mice have reduced neutrophil recruitment into their lungs and inflammatory mediators (19).

Studies using human airway cells have shown that bronchial epithelial cells stimulated with the viral mimetic dsRNA show increased expression of IL-36γ that was further enhanced by IL-17 (20). IL-36γ, but not IL-36α, was also induced in human peripheral blood mononuclear cells (PBMC) by the fungus *Aspergillus fumigatus* (21). This is important, as 37% of stable COPD subjects are colonized with *A*. *fumigatus* (22), potentially due to an inability of COPD macrophages to phagocytose these fungal spores (23). IL-36α, IL-36β, and IL-36γ all induce protein and gene expression of IL-6 and CXCL8 in normal human lung fibroblasts and bronchial epithelial cells, whereas exogenous addition of IL-36Ra reduces these mediators (24). This suggests that IL-36 cytokines may induce the release of these proinflammatory cytokines via NF-κB and MAPK signaling (24).

Elevated concentrations of IL-36 cytokines have been associated with a variety of chronic inflammatory diseases, including generalized pustular psoriasis (GPP), inflammatory bowel disease, rheumatoid arthritis, and systemic lupus erythematosus. Loss-of-function mutations within the IL-36Ra gene (*IL-36RN*) may result in GPP, an inflammatory skin disease characterized by elevated proinflammatory cytokines and immune cell and neutrophil infiltrates (25). Furthermore, there are promising data emerging from a completed phase I clinical trial using a neutralizing antibody targeting IL-36R in this disease (26). However, the role of the IL-36 family of cytokines has not been explored in COPD.

In the current study, we hypothesized that there is an altered expression of IL-36 cytokines in the COPD lung, and this imbalance drives the characteristic neutrophilic inflammation seen in this disease. We therefore examined the expression of IL-36 agonist and antagonist cytokines in the lungs of COPD subjects compared with healthy individuals. We studied the cellular source of IL-36 within the lung and identified potential effector cells of this family of proinflammatory cytokines. We also examined the mechanism by which IL-36 may amplify neutrophilic inflammation in the lungs of COPD subjects, and we highlight a critical role for these cytokines in perpetuating neutrophilic inflammation and progression in COPD.

## Results

### Increased IL-36 cytokines in COPD.

**Figure 1:**
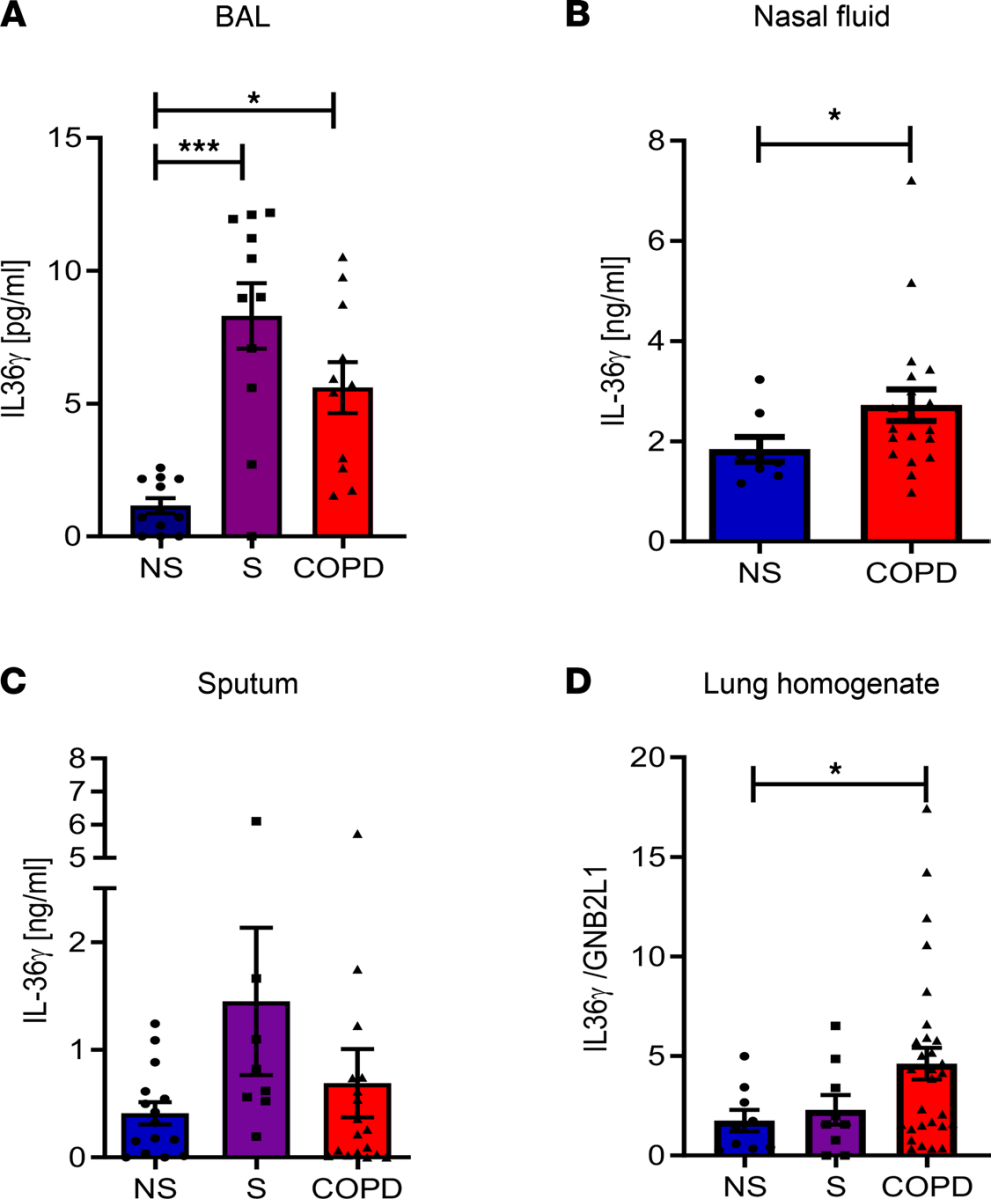
IL-36γ protein is elevated in COPD. (**A**–**C**) IL-36γ was measured in the bronchoalveolar lavage fluid (BALF) of nonsmokers (NS, ● blue, *n* = 12), smokers (S, ■ purple, *n* = 11), and patients with COPD (▲ red, *n* = 11) ; nasal fluid of NS (*n* = 8) and patients with COPD (*n* = 20); and sputum of NS (*n* = 15), smokers (*n* = 8), and patients with COPD (*n* = 18) by ELISA. (**D**) IL-36γ gene expression was examined in lung homogenate samples from NS (*n* = 9), smokers (*n* = 9), and patients with COPD (*n* = 29). Data are shown as mean ± SEM and analyzed by Kruskal-Wallis test with post hoc Dunn’s test (**A**, **C**, and **D**) or by Mann-Whitney *U* test (**B**); *P < 0.05, ***P < 0.001.

Firstly, IL-36 cytokines were examined in BALF from age-matched nonsmokers, smokers, and COPD subjects. Elevated concentrations of IL-36γ were found in both smoker and COPD subjects compared with nonsmokers (Figure 1A), whereas IL-36α and IL-36β were not detected (Supplemental Figure 1, A and B; supplemental material available online with this article; https://doi.org/10.1172/jci.insight.155581DS1). Increased release of IL-36γ was also measured in nasal secretions from COPD subjects compared with control subjects (Figure 1B), with concentrations being much higher (ng/mL) than the diluted BALF (pg/mL). No changes in IL-36α or IL-36β were observed in nasal fluid samples (Supplemental Figure 1, C and D), suggesting that IL-36γ is specifically upregulated in the airway mucosa. IL-36 cytokines are also reported to be increased in the serum (12) and sputum of COPD subjects (13). However, we found no significant increase in IL-36γ in sputum samples from smokers or COPD subjects (Figure 1C), although it was detectable and trended toward an increase. Neither IL-36α nor IL-36γ were altered in COPD serum samples, with most below the level of detection of the assay (Supplemental Figure 1, E and F). Gene expression was also examined in lung homogenate samples from nonsmokers, smokers, and COPD subjects. *IL-36G* mRNA was significantly upregulated in COPD subjects (Figure 1D).

### Cellular source of IL-36 in the lung.

**Figure 2:**
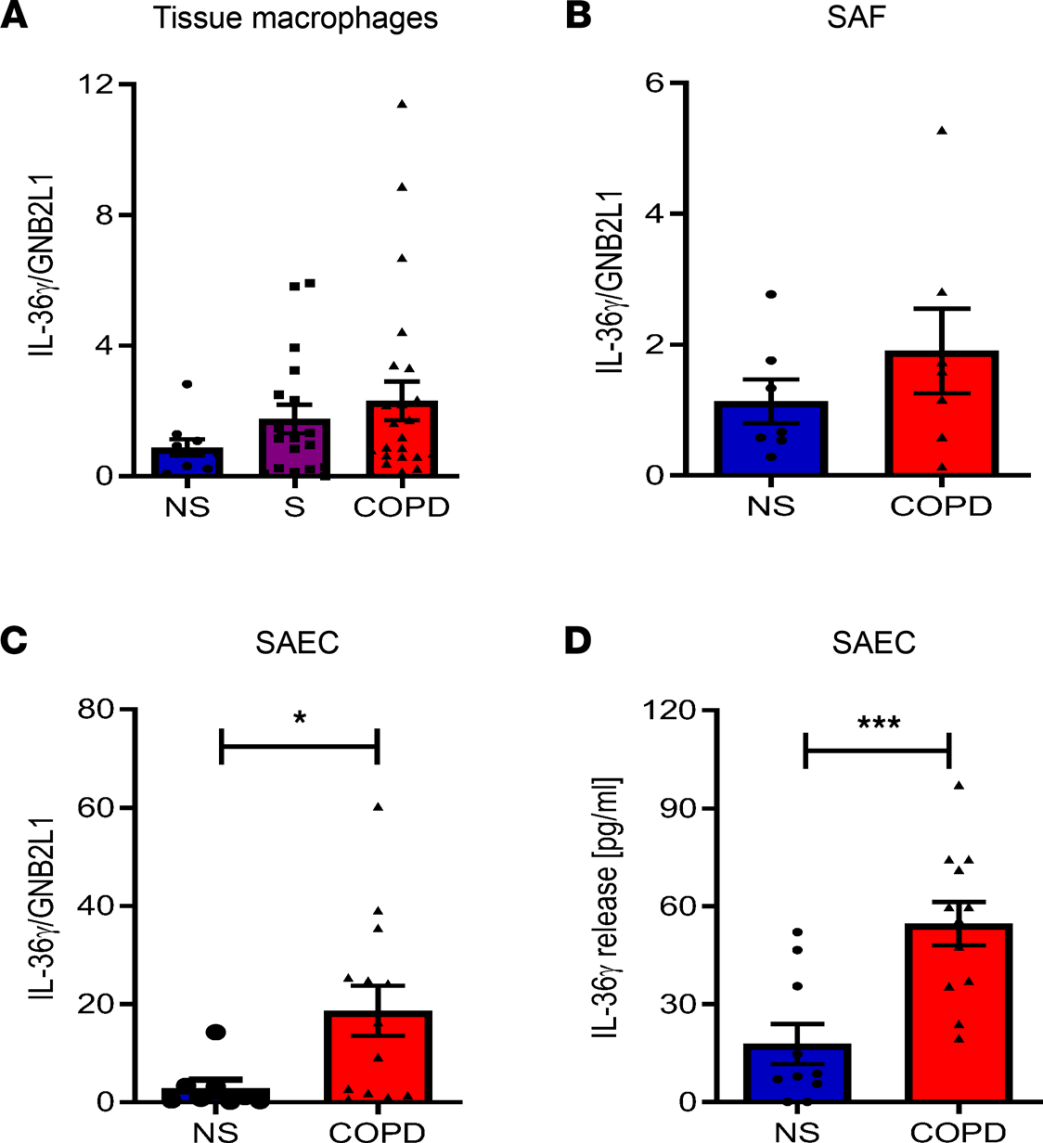
Small airway epithelial cells express and release IL-36γ in COPD. (**A**–**C**) Gene expression of *IL-36γ* was measured in lung tissue-derived macrophages (TMφ), small airway fibroblasts, and small airway epithelial cells from nonsmokers (NS, *●* *n* = 7–10), smokers (S, ■ *n* = 18), and patients with COPD (▲, *n* = 7-24). (**D**) IL-36γ release from small airway epithelial cells from NS (*n* = 10) and COPD (*n* = 12) patients, measured by ELISA. Data are shown as mean ± SEM and analyzed by Kruskal-Wallis test with post hoc Dunn’s test (**A**) or by Mann-Whitney *U* test (**B**–**D**); *P < 0.05, ***P < 0.001.

To determine the cellular source of elevated IL-36γ within the airways of COPD subjects, gene expression of IL-36 cytokines was examined in key cells involved in COPD pathogenesis: lung tissue-derived macrophages (TMφ), small airway fibroblasts (SAF), and small airway epithelial cells (SAEC). TMφ from COPD subjects displayed a nonsignificant trend toward increased *IL-36G* expression in cells from smokers and COPD subjects compared with controls (Figure 2A). Expression of *IL-36A* was unchanged between patient groups, whilst *IL-36B* RNA was significantly downregulated in smokers but unchanged in COPD subjects compared with nonsmokers (Supplemental Figure 2, A and B). *IL-36A* expression was not detected in SAF, and there was no difference in gene expression of *IL-36G* between COPD SAF and nonsmokers (Figure 2B), with a similar pattern for *IL-36B* (Supplemental Figure 2C). In contrast, *IL-36G* expression was significantly increased in COPD SAEC compared with nonsmokers (Figure 2C), with a significant increase in *IL-36A* expression but no change in *IL-3B* expression (Supplemental Figure 2, D and E). IL-36γ protein release from TMφ and SAF was below the level of detection. However, IL-36γ release from SAEC was detectable, and COPD SAEC released significantly more IL-36γ compared with nonsmoking controls (Figure 2D), suggesting that airway epithelial cells could be a major source of IL-36γ in peripheral airways, under basal conditions, and that these cells may be responsible for the elevated levels observed in BALF and nasal secretions in COPD.

### IL-36Ra is reduced in COPD.

IL-36 signaling can be modulated by binding of IL-36Ra to the IL-36R, thereby suppressing receptor activation (7). Genetic mutations in the IL-36Ra can drive disease, with a loss-of-function mutation in IL-36Ra being a likely mechanism in GPP (25). These data suggest not only the importance of the upregulation of IL-36 agonists, but also the modulation of the IL-36 receptor antagonist. The levels of IL-36Ra in the context of COPD have not previously been reported.

**Figure 3:**
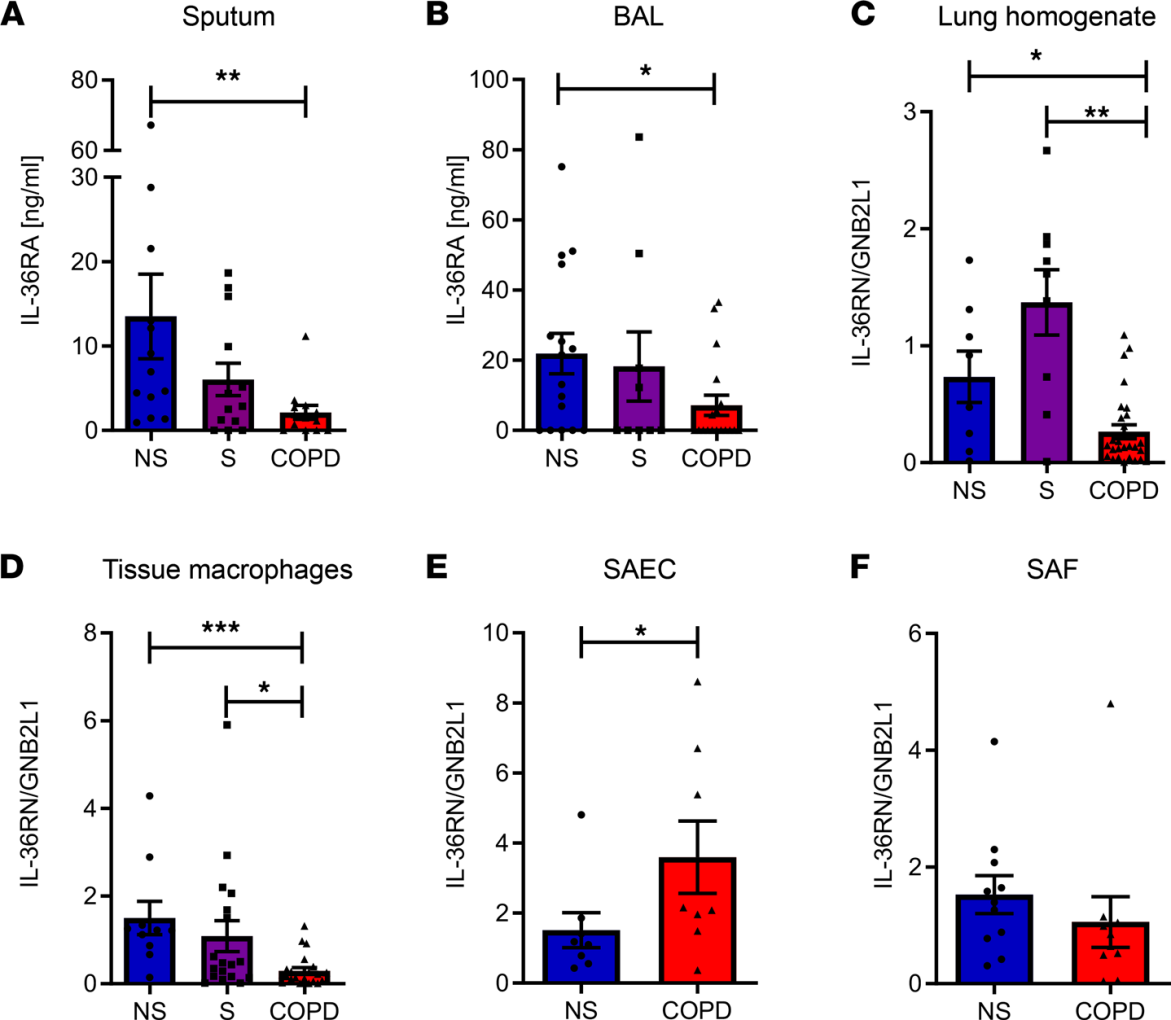
IL-36 receptor antagonist is reduced in patients with COPD. (**A** and **B**) IL-36Ra protein was detected in the BALF and sputum of nonsmokers (NS, ● *n* = 13-16), smokers (S, ■ *n* = 9-13), and COPD (▲ *n* = 13-18) patients by ELISA. (**C**–**F**) Gene expression of IL-36RN was detected in lung homogenate samples from NS (*n* = 9), smokers (*n* = 9), patients with COPD (*n* = 29); lung tissue-derived macrophages; small airway epithelial cells; and small airway fibroblasts. NS (*n* = 5–10), smokers (*n* = 7–18), and patients with COPD (*n* = 7–24). Data are shown as mean ± SEM and analyzed by Kruskal-Wallis test with post hoc Dunn’s test (**A**–**D**) or by Mann-Whitney *U* test (**E** and **F**); *P < 0.05, **P < 0.01, ***P < 0.001.

The protein levels of IL-36Ra were significantly reduced in BAL and sputum of COPD subjects compared with nonsmokers (Figure 3, A and B). IL-36Ra gene expression (*IL-36RN*) was also significantly reduced in COPD lung homogenate samples compared with nonsmokers and smokers (Figure 3C), with smokers showing a trend toward increased *IL-36RN* expression, potentially as a protective mechanism.

To determine the cellular source of IL-36Ra, we examined gene expression of *IL-36RN* in different cells. *IL-36RN* was significantly downregulated in TMφ from COPD subjects compared with both nonsmoker and smoker samples (Figure 3D); this appeared to be specific for the receptor antagonist of IL-36, as this was not seen for IL-1 receptor antagonist (Supplemental Figure 2F). SAEC displayed a significant increase in *IL-36RN* in cells from COPD subjects (Figure 3E), while there was no change in expression of *IL-36RN* in SAF between nonsmoker and COPD subjects (Figure 3F). Gene expression of the other IL-36 antagonist, IL-38, was undetectable in all cell types examined. Since changes in IL-36R expression may also alter IL-36 signaling, the gene expression of the IL-36R was examined and found to be unchanged in all patient groups — in TMφ, SAEC, and SAF (Supplemental Figure 2, G–I). These data suggest that, along with elevated levels of IL-36γ in patients with COPD, there is dysregulation of the receptor antagonist that may amplify the effects of IL-36 on the lung.

### IL-36γ induction.

**Figure 4:**
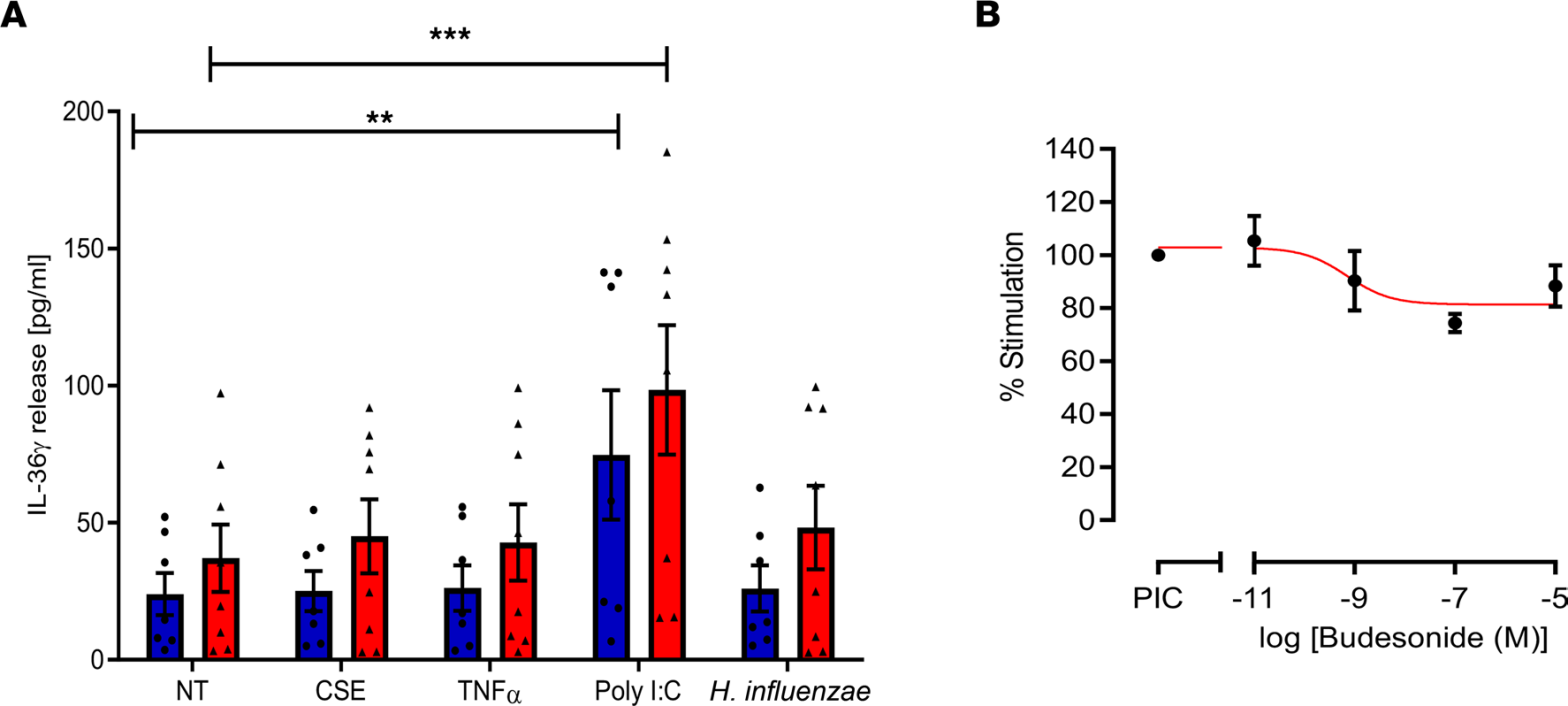
IL-36γ protein release is driven by steroid insensitive TLR3 activation. (**A**) Small airway epithelial cells from nonsmokers (blue bars ●, *n* = 7) and COPD subjects (red bars ▲, *n* = 8) were exposed to media alone (no treatment, NT), 10% (v/v) cigarette smoke extract (CSE), 10 ng/mL TNF-α, 100μg/mL poly(I:C), or 1.5 × 10^10^ CFU/mL *H*. *influenzae* (HI) for 24 hours. Media was collected and IL-36γ release was measured by ELISA. (**B**) Small airway epithelial cells from *n* = 4 nonsmokers were treated with 100 μg/mL poly(I:C) in the presence or absence of increasing concentrations of budesonide for 24 hours. Media was collected, and IL-36γ was measured by ELISA. Data are shown as mean ± SEM and analyzed by 2 way ANOVA with post hoc Dunnett’s multiple-comparison test; **P < 0.01, ***P < 0.001 versus NS.

Since SAEC appear to be the main source of IL-36γ, we investigated whether SAEC could be stimulated to release more IL-36γ in response to stimuli that may exacerbate COPD. We showed that many of the common airway epithelium stimulants, such as cigarette smoke extract (CSE), TNF-α, or the common colonizing respiratory pathogen *Haemophilus influenzae* failed to stimulate IL-36γ release (Figure 4A). However, the TLR3 agonist poly(I:C) stimulated release of IL-36γ from SAEC (Figure 4A) and in a concentration-dependent manner (Supplemental Figure 3A), with cells from COPD subjects releasing more because of a higher baseline release of this cytokine. Still, poly(I:C) failed to induce further release of IL-36α (Supplemental Figure 3B), and IL-36β protein was undetectable in these cells. The same stimuli were applied to nonsmoker and COPD SAF, with nonsmoker SAF being unresponsive to all stimuli, while COPD SAF released more IL-36γ in response to all stimuli, although these were at a lower level when compared with SAEC (Supplemental Figure 3C). These data suggest that COPD SAEC may respond to viral infection by releasing IL-36γ, thereby perpetuating the already-elevated levels of IL-36γ in the lung.

### Effect of glucocorticosteroids.

Since glucocorticosteroids are often given as an antiinflammatory treatment to many COPD subjects, particularly during viral-induced exacerbations, we examined whether TLR3 induction of IL-36γ was glucocorticosteroid sensitive. However, increasing concentrations of the glucocorticosteroid budesonide had little effect on poly(I:C)-stimulated release of IL-36γ from SAEC (Figure 4B). These data suggest that treatment of a viral exacerbation with corticosteroids will not suppress the elevated levels of IL-36γ that may be induced during a viral infection.

### Effects of IL-36γ on lung cells.

**Figure 5:**
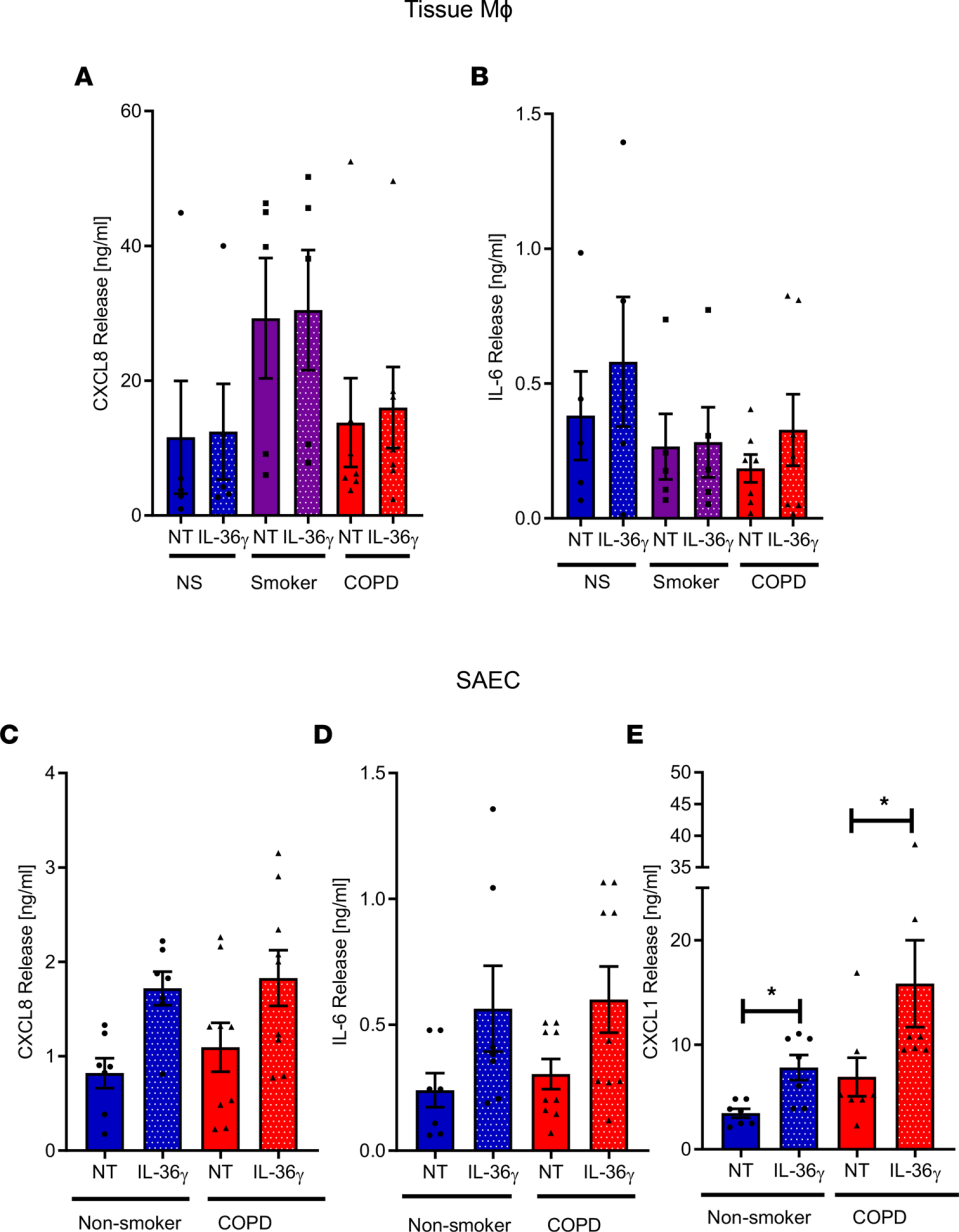
Effect of IL-36γ on lung tissue macrophages and small airway epithelial cells. Lung tissue–derived macrophages from nonsmokers (*n* = 4), smokers (*n* = 5), or patients with COPD (*n* = 7) were incubated in the absence (NT) or presence of 100 ng/mL IL-36γ for 24 hours. (**A** and **B**) Media was harvested and release of CXCL8 and IL-6 were measured by ELISA. Small airway epithelial cells from nonsmokers (*n* =7) or patients with COPD (*n* = 7-9) were incubated in the absence (NT) or presence of 100 ng/mL IL-36γ for 24 hours. (**C**–**E**) Media was harvested and release of CXCL8, IL-6, and CXCL1 were measured by ELISA. Data are shown as mean ± SEM and analyzed by Kruskal-Wallis test with post hoc Dunn’s test; *P < 0.05.

IL-36γ activates multiple different cell types (7). Therefore, we investigated which pulmonary cells are the likely targets in COPD. TMφ, SAEC, and SAF from COPD subjects and controls were stimulated with activated IL-36γ, and release of CXCL1 (GRO-α), CXCL8 (IL-8), IL-6, and granulocyte-macrophage CSF (GM-CSF) were measured, as they are all are increased in COPD lungs. Nonsmoker, smoker, and COPD TMφ were unresponsive to IL-36γ, with no change in CXCL8 and IL-6 release (Figure 5, A and B), and GM-CSF was undetectable. Stimulation of SAEC with IL-36γ led to a modest increase in both CXCL8 and IL-6 (Figure 5, C and D). However, there was a significant increase in CXCL1 release from both nonsmoker and COPD SAEC (Figure 5E).

**Figure 6:**
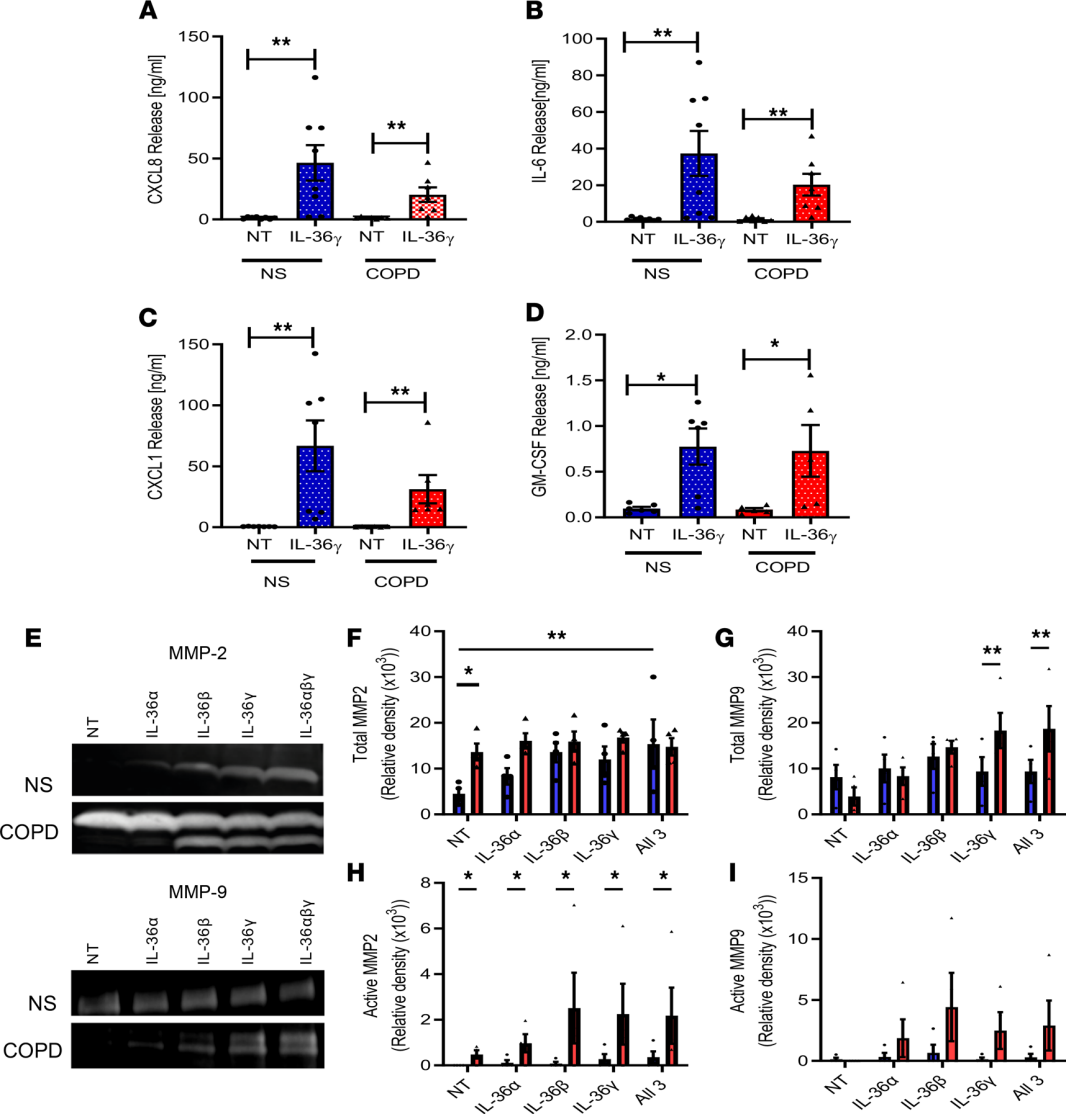
IL-36γ activates small airway fibroblasts, leading to chemokine and protease release. Small airway fibroblasts from nonsmokers (*n* = 8) and patients with COPD (*n* = 7) were cultured in the absence (NT) or presence of 100 ng/mL IL-36γ for 24 hours. (**A**–**D**) Media was harvested and CXCL8, IL-6, CXCL1, and GM-CSF were measured by ELISA. Small airway fibroblasts from nonsmokers (*n* = 3) and patients with COPD (*n* = 3) were cultured in the absence (NT) or presence of 33 ng/mL IL-36α, IL-36β, IL-36γ, or all 3 in combination. (**E**) Media was collected and zymography performed. (**F**–**I**) Relative density of total MMP2 (**F**) and MMP9 (**G**) and active MMP2 (**H**) and MMP9 (**I**). Data are shown as mean ± SEM and analyzed by Kruskal-Wallis test with post hoc Dunn’s test; *P < 0.05, **P < 0.01

In contrast to the data derived from TMφ and SAEC, stimulation of SAF with IL-36γ induced a large and significant increase in the release of CXCL8, IL-6, CXCL1, and GM-CSF (Figure 6, A–D), with no difference between control and COPD cells. The magnitude of cytokine release was some 25-fold higher than cytokine release from SAEC. These data strongly suggest that SAF compose a key effector cell type for IL-36γ in the airways. Furthermore, these proinflammatory cytokines and chemokines are elevated in patients with COPD (27–29) and may be responsible for neutrophil recruitment into the airways, which is a key feature of this disease. To examine whether these effects were specific to IL-36γ or if there was an additive effect of all 3 cytokines in combination, all cell types were treated with the individual IL-36 isoforms or in combination. CXCL8 release from SAF, SAEC, and TMφ was the same for each isoform and when in combination; SAF still released the greatest levels of cytokines in response (Supplemental Figure 4, A–C). These data again strongly suggest that SAF are a potential effector cell type for IL-36γ and may have a key role in neutrophil recruitment observed in this disease via increased neutrophil chemokine release.

SAF are found at the site of airways obstruction in COPD and are important in the development of peribronchiolar fibrosis, which is the hallmark of early disease (30). Matrix metalloproteinases 2 (MMP2) and MMP9 are increased in COPD and are highly activated (31, 32). SAF from COPD subjects release increased levels of active MMP2 (4.6-fold) and total MMP9 (4.7-fold) when stimulated with IL-36γ (Figure 6, E–I), and stimulation of these cells with any of the IL-36 isoforms appears to lead to an increase in activation of MMP2 (Figure 6E). Taken together, these data highlight the potential role of SAF in driving neutrophil recruitment and airway remodeling in COPD.

Fibroblast phenotypes are also altered in COPD, with differentiation toward myofibroblasts, leading to greater collagen deposition within the lung (33). We, therefore, examined the effect of IL-36γ on these fibrosis markers in comparison with IL-1α to determine whether other members of the IL-1 family elicited similar effects. *COL1A1* and *COL3A1* gene expression appeared unchanged in SAF from both nonsmoker and COPD subjects when treated with either IL-36γ or IL-1α (Supplemental Figure 5, A and B). However, *α-SMA*, a myofibroblast differentiation marker, was significantly decreased in SAF from both nonsmoker and COPD subjects by both IL-36γ and IL-1α (Supplemental Figure 5C), suggesting that IL-36γ was driving these cells away from a myofibroblast phenotype to a potentially more proinflammatory and proteolytic state. This was confirmed by an increase in CXCL8, IL-6, and MMP9 gene expression in these cells (Supplemental Figure 5, D–F). Interestingly, as seen previously with protein release, SAEC stimulated with IL-36γ did not respond as robustly as fibroblasts, with a small but significant change in the gene expression of CXCL8 and IL-6, but no change in MMP2 or MMP9 (Supplemental Figure 5, I–L).

IL-36Ra may be induced by IL-36 cytokines as a negative feedback loop. We, therefore, also examined the effect of IL-36γ and IL-1α on gene expression of IL-36RN. We observed increased expression of *IL-36RN* in response to IL-36γ in nonsmoker SAF, but this was blunted in COPD SAF (Supplemental Figure 5G). Similarly, IL-1α caused a significant increase *IL-36RN* in nonsmoker SAF, but this was not seen in COPD SAF (Supplemental Figure 5G). Interestingly, *IL-1RA* was induced in both nonsmoker and COPD SAF when stimulated with IL-1α, but when stimulated with IL-36γ, the response was again blunted in COPD subjects (Supplemental Figure 5H). Additionally, IL-36γ did not stimulate expression of TGF-β or SMAD3 in SAF (Supplemental Figure 6), suggesting that IL-36γ does not alter SAF phenotype. This suggests that IL-36γ–mediated upregulation of IL-36RN is attenuated in COPD fibroblasts, resulting in increased activity of IL-36γ.

### Effect of glucocorticosteroids on IL-36γ responses.

**Figure 7:**
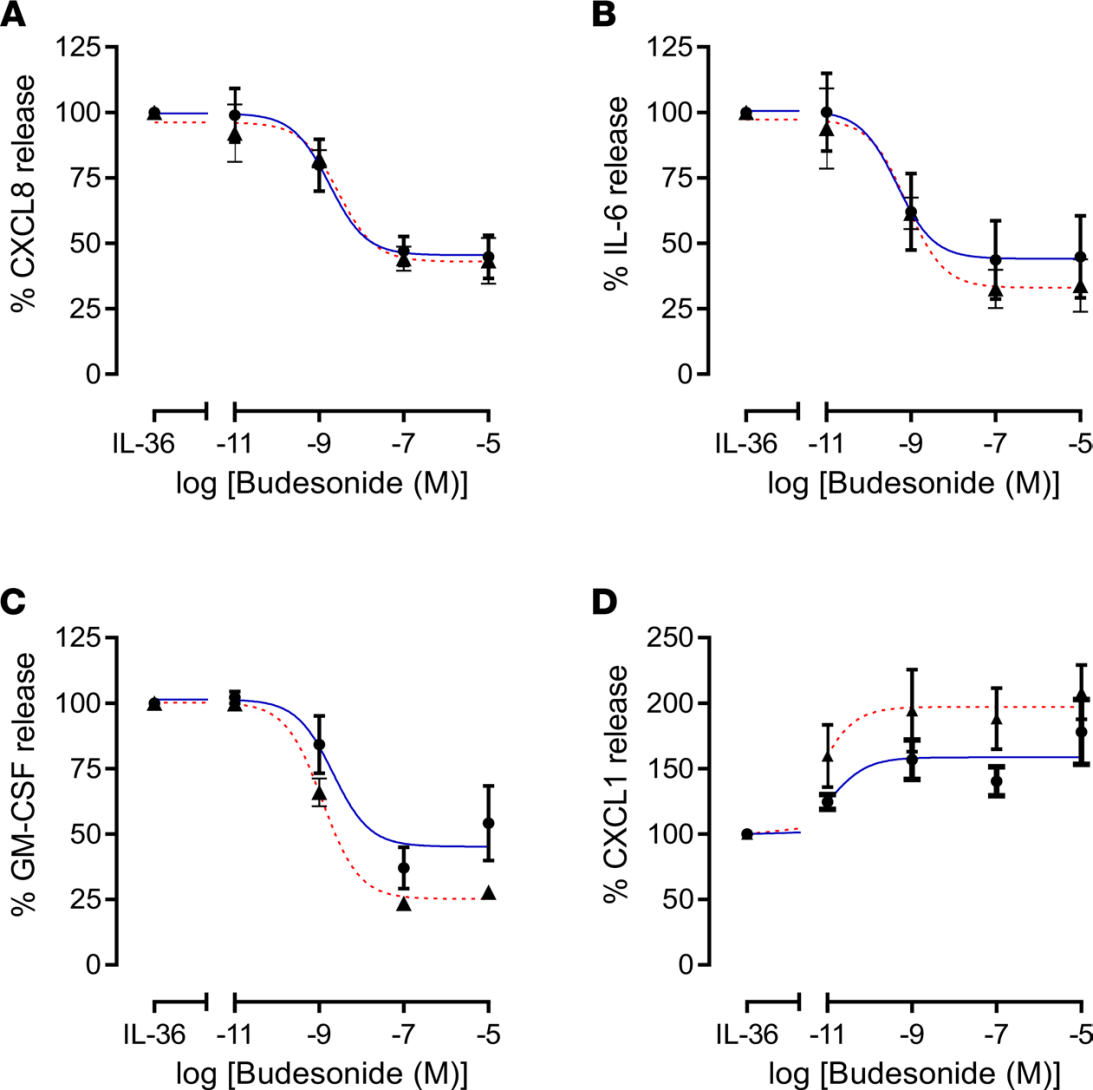
IL-36 stimulation of SAF is glucocorticosteroid sensitive, except the neutrophil chemokine CXCL1, which is induced by budesonide. Small airway fibroblasts from nonsmokers (● *n* = 4) or COPD (▲ *n* = 4) were treated with active IL-36γ for 24 hours in the absence or presence of budesonide at varying concentrations. (**A**–**D**) CXCL8, IL-6, GM-CSF, and CXCL1 release were measured by ELISA. Data are presented as mean ± SEM.

Since inhaled glucocorticosteroids are commonly used in the treatment of COPD, we examined whether IL-36γ–driven inflammation was steroid sensitive. SAF from nonsmokers and COPD subjects were stimulated with IL-36γ, and the effect of increasing concentrations of budesonide on the release of the chemokines and cytokines was examined. Interestingly, CXCL8, IL-6, and GM-CSF were all reduced by budesonide in a concentration-dependent manner (Figure 7, A–C). However, CXCL1, one of the major neutrophil-recruiting chemokines in COPD, was surprisingly increased by budesonide treatment (Figure 7D), suggesting that steroid treatment may paradoxically further perpetuate IL-36γ inflammation by increasing neutrophil recruitment.

### Serine proteases activate IL-36γ.

**Figure 8:**
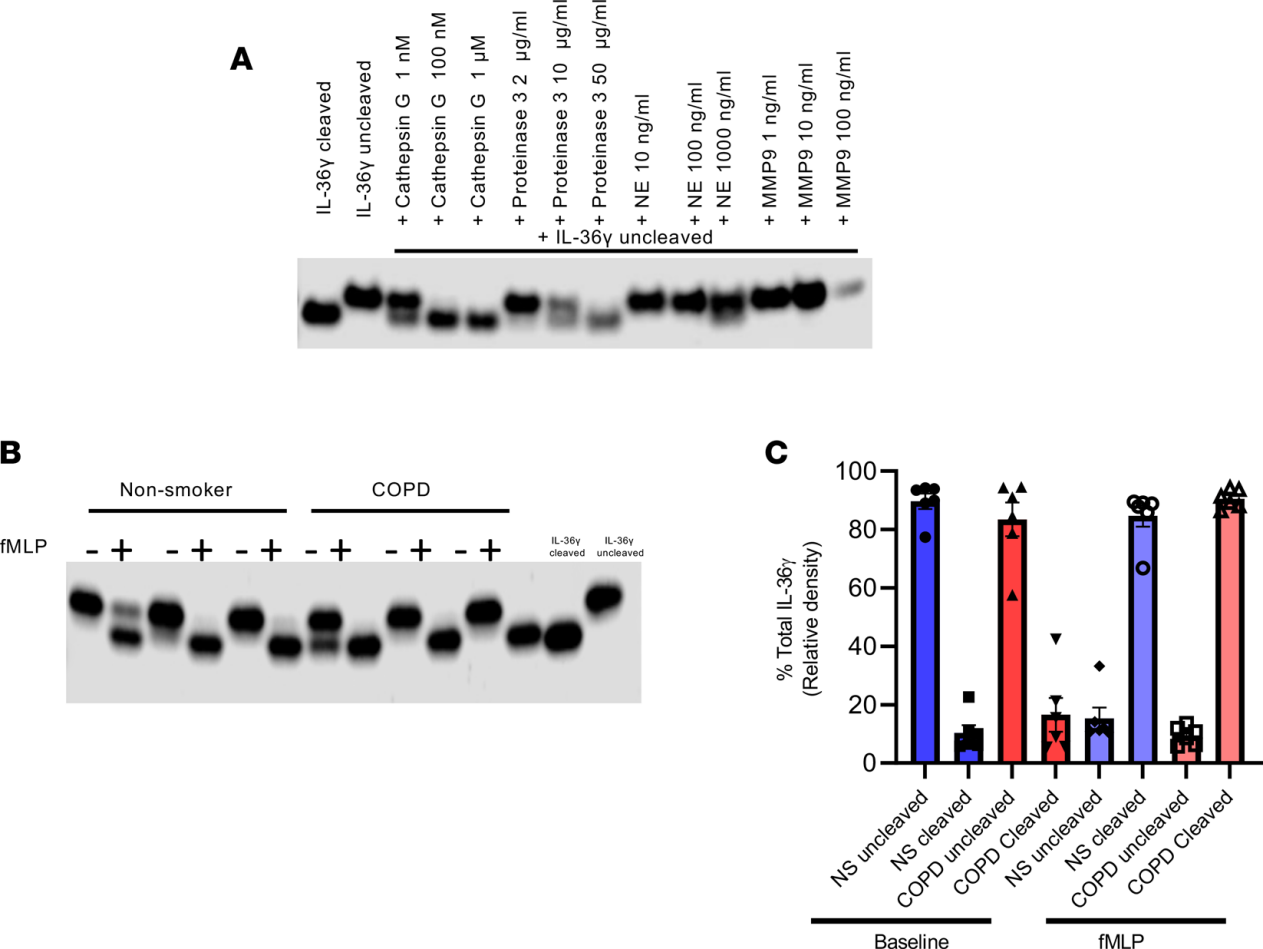
Effect of neutrophil serine proteases on activation of IL-36γ. (**A**) Recombinant full length IL-36γ was incubated with cathepsin G, proteinase-3, neutrophil elastase (NE), or MMP9 at varying concentrations for 2.5 hours, and Western blot analyses were performed to separate inactive form and active (cleaved) IL-36γ. (**B**) Neutrophils from nonsmokers (*n* = 6) and patients with COPD (*n* = 6) were left at baseline or activated with fMLP, and the supernatant was collected. Supernatants were incubated with recombinant full-length IL-36γ for 2.5 hours, and Western blot analyses were performed to separate inactive form and active (active) IL-36γ (data shown are representative blots). (**C**) Optical density of bands was performed, and data are presented as mean ± SEM.

Previous studies have suggested that intratracheal instillation of IL-36 into the lungs of mice induces neutrophil recruitment (19, 34, 35). Recruitment and subsequent activation of neutrophils at the site of inflammation in the lung leads to the release of proteolytic enzymes, such as the serine proteases neutrophil elastase, proteinase-3, and cathepsin G, all of which are elevated in the COPD lung (10). Elevated numbers of neutrophils and macrophages are found within the lungs of patients with COPD (2), with both cell types releasing proteases, which — when unchecked — can lead to damage and remodeling of the lung (36). Neutrophil elastase, proteinase-3, and cathepsin G have all been suggested to cleave IL-36 cytokines, although there are conflicting results within the literature (8, 9, 37). We therefore assessed whether neutrophil proteases (neutrophil elastase, proteinase-3, and cathepsin G) or a macrophage protease (MMP9) could cleave and activate IL-36γ. Utilizing a cell-free assay, we incubated recombinant full-length IL-36γ with various concentrations of these proteases. Our results show that both cathepsin G and proteinase-3 were capable of cleaving IL-36γ into its active form, whereas neutrophil elastase did not (Figure 8A), suggesting that neutrophils may activate IL-36γ in COPD.

Since it appeared that neutrophil proteases cleaved IL-36γ, we next sought to see whether activated neutrophil products from both nonsmokers and COPD subjects could cleave and activate IL-36γ. Full-length IL-36γ was converted to the active form by both nonsmoker and COPD N-Formylmethionine-leucyl-phenylalanine–activated (fMLP-activated) neutrophils, although data suggest that basally released COPD neutrophil products may be able to cleave IL-36γ (Figure 8, B and C). Elevated numbers of neutrophils within the COPD lung may, therefore, have the capability to further activate IL-36 cytokines; further amplifying the inflammation that IL-36γ may cause within the COPD lung.

### Blocking the IL-36R prevents inflammatory crosstalk between COPD SAEC and SAF.

**Figure 9:**
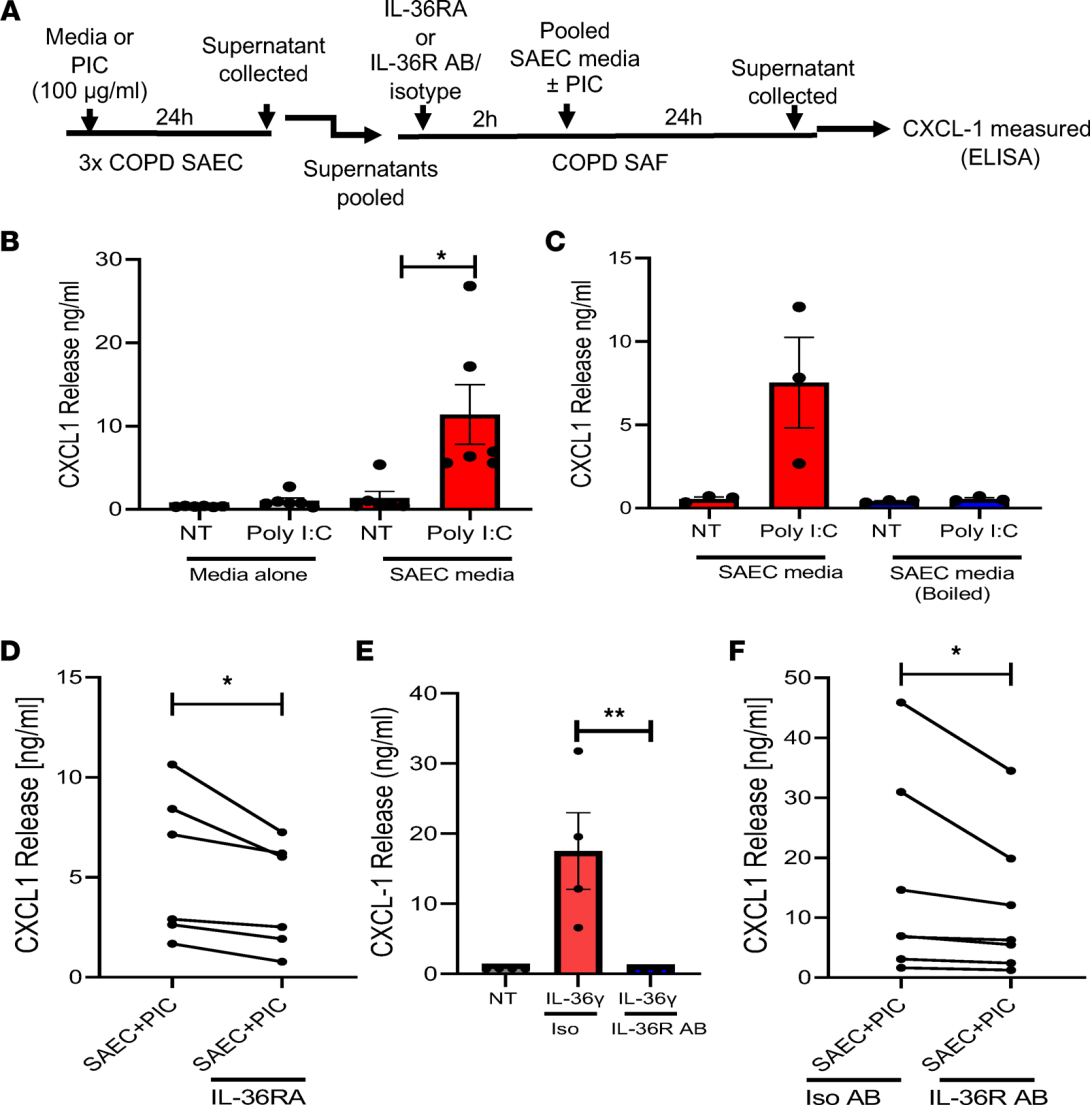
Optimization of therapeutic IL-36R inhibition experiments. (**A**) Schematic of experimental procedure. (**B**) Small airway fibroblast (*n* = 6) were treated with diluted media, media + poly(I:C), conditioned media alone from SAEC, or conditioned media from poly(I:C)–treated SAEC (ranging from 10- to 200-fold) for 24 hours (pooled media from 3 patients with COPD). CXCL1 levels were then measured. (**C**) Small airway fibroblasts (*n* = 4) were treated with diluted (ranging from 10- to 200-fold) conditioned media alone from SAEC or conditioned media from poly(I:C)–treated SAEC that had and hadn’t been boiled for 24 hours. CXCL1 levels were then measured. (**D**) Small airway fibroblast (*n* = 6) were treated with diluted (ranging from 10- to 200-fold) conditioned media alone from SAEC or conditioned media from poly(I:C)–treated SAEC (ranging from 10- to 200-fold) with or without pretreatment for 2 hours with recombinant IL-36Ra (100 ng/mL). CXCL1 levels were then measured. (**E**) Small airway fibroblast (*n* = 4) were pretreated for 2 hours with isotype control (IgG1) antibody or a IL-36R neutralizing antibody and then treated with 100 ng/mL of IL-36γ for 24 hours. (**F**) Small airway fibroblasts (*n* = 7) were treated with diluted (ranging from 10- to 200-fold) conditioned media alone from SAEC or conditioned media from poly(I:C)–treated SAEC with pretreatment with either isotype control (IgG1) antibody or a IL-36R neutralizing antibody for 2 hours. CXCL1 levels were then measured. Data are presented as mean ± SEM analyzed by either Kruskal-Wallis with post hoc Dunn’s test (**B**, **C**, and **E**) or Wilcoxon matched-pairs signed-rank test (**D** and **F**); *P < 0.05, **P < 0.01.

Since we have shown that IL-36Ra is downregulated in COPD subjects and may exacerbate the inflammatory response of IL-36 cytokines in the COPD lung, we investigated whether reintroduction of IL-36Ra could inhibit IL-36γ–driven inflammation. Experiments were devised whereby media from poly(I:C)-stimulated SAEC from COPD subjects was transferred to COPD SAF, and CXCL1 was measured as an output. A schematic of the experiment is depicted in Figure 9A. The transferred media contained IL-36γ (unstimulated, 15.9 pg/mL; stimulated, 307.9 pg/mL) and CXCL1 (unstimulated, 1.9ng/mL; stimulated, 3.1 ng/mL). Native media from poly(I:C)-stimulated SAEC led to a significant release of CXCL1 from SAF as previously seen, while little CXCL1 release was seen when SAF were stimulated with poly(I:C) alone (Figure 9B). To show that the induction to CXCL1 in the SAF was a consequence of a protein mediator within the media, the media was boiled to denature proteins, and SAF were treated with this media (Figure 9C). Transfer of this conditioned media also increased production of CXCL8 and IL-6 (Supplemental Figure 7). Having established a media transfer system, SAF were then treated for 2 hours with 100 ng/mL of recombinant IL-36Ra, before being treated with SAEC media. Pretreatment with IL-36Ra led to a significant reduction in CXCL1 release from SAF treated with poly(I:C)-stimulated SAEC media (Figure 9D). To confirm these findings, we utilized an IL-36R neutralizing antibody. SAF from COPD subjects were treated with 50 μg/mL of either isotype control or an antibody that binds the IL-36R and blocks signaling, followed by 100 ng/ mL of IL-36γ. IL-36γ induced CXCL1 release from cells treated with the isotype control, but this was abolished in cells pretreated with the IL-36R neutralizing antibody, showing that the antibody inhibited IL-36–mediated CXCL1 release (Figure 9E). SAF were then treated for 2 hours with the isotype control or IL-36R antibody, before being treated with SAEC media. Pretreatment with IL-36R led to a significant reduction in CXCL1 release from SAF treated with poly(I:C)-stimulated SAEC media (Figure 9F). These data suggest that blocking IL-36R by increasing the reduced endogenous levels of IL-36Ra or by directly blocking the IL-36R using a neutralizing antibody make it possible to prevent the crosstalk between virally stimulated COPD SAEC and COPD SAF. These data also suggest that blocking IL-36 signaling via these 2 methods may reduce virally induced IL-36–mediated inflammation in COPD.

## Discussion

**Figure 10:**
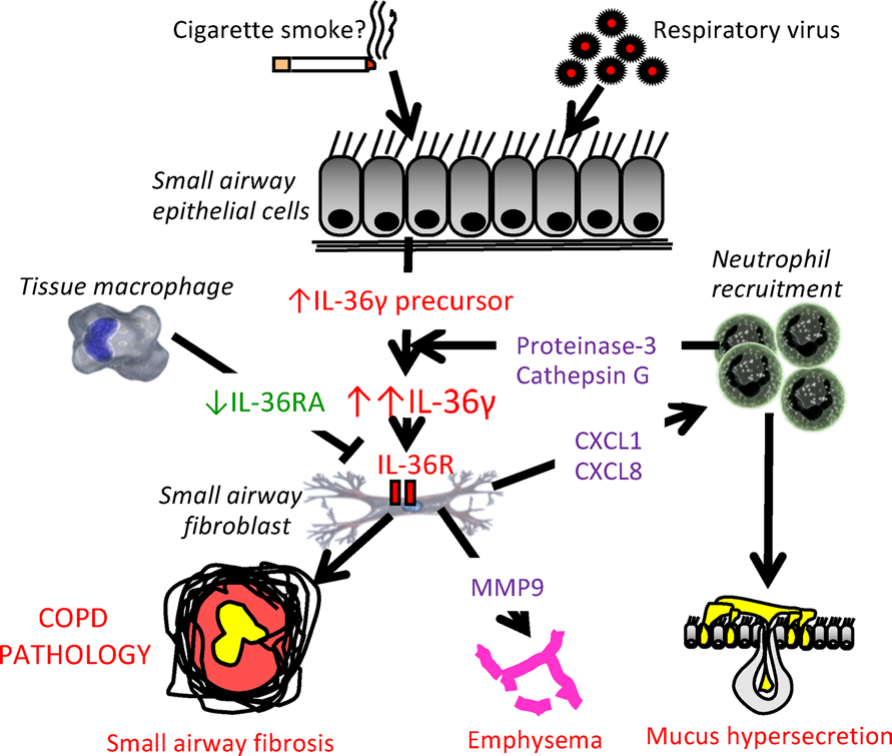
Schematic outlining the role of IL-36γ in COPD pathophysiology. Small airway epithelial cells are a major source of IL-36γ in the COPD lung, and the release of IL-36γ is elevated at baseline in these patients. These elevated levels can be exacerbated by viral infection, which may be perpetuated in those who smoke. The resultant pro–IL-36γ is cleaved by neutrophil-derived proteases such as cathepsin G and proteinase-3, generating the active form of IL-36γ. This acts on small airway fibroblasts, leading to the release of MMP2 and MMP9, as well as leading to expression of the chemokines CXCL1 and CXCL8. CXCL1 and CXCL8 recruit neutrophils and perpetuate the cycle of neutrophilic inflammation. This process is amplified in patients with COPD due to the loss of the endogenous IL-36 receptor, IL-36Ra, from macrophages. The released elastases and MMPs contribute to all 3 pathophysiological features of COPD, small airway remodeling, emphysema, and mucous hypersecretion.

Neutrophilic inflammation is characteristic of COPD airways and is associated with increased expression of neutrophil chemoattractants CXCL1 and CXCL8 (27, 38). Our study describes a mechanism for the amplification and perpetuation of chronic neutrophilic inflammation in COPD. We confirm previous findings that IL-36γ — but, in this study, not IL-36α — is elevated in the lungs of COPD subjects, but we show for the first time to our knowledge that there are reduced levels of IL-36Ra, suggesting enhanced IL-36 signaling in COPD lungs. Examining multiple cell types from nonsmokers, smokers, and COPD subjects we established that elevated levels of IL-36γ in COPD BALF and nasal fluid is potentially derived from epithelial cells, which release higher basal IL-36γ levels than cells from nonsmokers. We show that SAF appear to be the potential IL-36γ effector cell, releasing marked amounts of chemokines, proinflammatory cytokines, and proteases upon stimulation. These recruit neutrophils into the lung that may activate the elevated IL-36γ by releasing serine proteases capable of cleaving IL-36γ into its active form; our data suggest that these proteases are cathepsin G and proteinase-3, but not neutrophil elastase. We show that treating SAF with IL-36Ra or using a therapeutic antibody that blocks IL-36R–mediated signaling inhibits viral-induced IL-36–mediated inflammatory crosstalk between SAEC and SAF in COPD. IL-36γ may, therefore, drive COPD pathophysiology via the recruitment and activation of neutrophils into the lung, leading to small airway remodeling, emphysema, and mucus hypersecretion (Figure 10).

IL-36γ was the only IL-36 family agonist detected in BALF, and levels were elevated in both smokers and COPD subjects, although one caveat is that the ELISA kit used may not distinguish between active and inactive IL-36γ. Previously, Kovach et al., reported that IL-36α, as well as IL-36γ, may also be elevated in the BALF of COPD subjects and smokers and, in agreement with our study, that IL-36β was undetectable. This may be due to smoking, as their cohort (12) included current smokers, whereas all of our COPD subjects were ex-smokers. Furthermore, exposure of SAEC to CSE in vitro did not stimulate release of IL-36γ in our study. This again contrasts with Kovach et al., who found increased release of IL-36γ in response to CSE but may reflect differential responses to CSE by epithelial cells from peripheral airways. Alternatively, this may reflect differences between chronic and acute exposure. Others have reported increased *IL-36*γ mRNA expression in response to CSE in bronchial epithelial cells but only after 8 hours, and it was reduced 2-fold after 24 hours stimulation (39). Although smoking may affect IL-36γ release, it appears that this alone is unlikely to account for the elevated basal release of COPD SAEC.

Reduced expression of IL-36Ra in COPD subjects was clear, and this may amplify and perpetuate IL-36–mediated inflammation within the lungs of COPD subjects compared with those who smoke. IL-36Ra binds to the IL-36R with a greater affinity and for a greater duration than IL-36 agonists, suggesting that its loss may be detrimental in overcoming IL-36 agonist–mediated inflammation (40). This is pertinent, as we were unable to detect IL-38, another antagonist of IL-36R. However, the effect of patient treatment on expression of IL-36Ra is not known and may impact expression. Similarly, the lung tissue used in this study was obtained from patients undergoing tumor resection, and this may alter expression of IL-36Ra; however, expression of this protein increases in other cancers (41, 42). Loss of IL-36Ra is prevalent in GPP, where missense mutation in the *IL-36RN* gene leads to IL-36Ra deficiency and drives this disease (25). There are no indications to date of a similar genetic defect in COPD; however, clinical trials are underway in GPP, assessing whether targeting the IL-36R with a blocking monoclonal antibody (Spesolimab) prevents and reverses this disease (26). Our data suggest that the effects of elevated levels of IL-36γ in COPD are likely to be greatly amplified by the reduced secretion of IL-36Ra and that blocking IL-36R may be of clinical benefit.

IL-1 is highly abundant in all cell types, but IL-36–related cytokines appear to be more cell specific, with an expression profile suggesting induction in epithelial cells (43). When examining the source of IL-36γ in the lung, we detected IL-36γ release only from SAEC, with levels being undetectable in SAF and TMφ, suggesting the epithelium as a major source. Previous data have also suggested that bronchial epithelial cells release IL-36 cytokines in response to dsRNA. Our data using poly(I:C) confirm these findings and again showed no release from TMφ and SAF with this stimulus.

The epithelium is the major site of viral infection within the lung (44), and our data suggest that IL-36γ can be further induced in COPD SAEC when treated with the viral mimetic poly (I:C). Upper respiratory tract viral infections are major causes of COPD exacerbations and leading causes of hospitalization for patients with COPD (45). Our data suggest that IL-36γ could be a potential biomarker for viral infections in COPD, especially as IL-36 cytokines can be detected in nasal samples; therefore, patients can be noninvasively tested during these episodes. Acute exacerbations of COPD are also triggered by bacterial infections, particularly *Haemophilus influenzae,* but treatment of SAEC with *H*. *influenzae,* had no effect on the release of IL-36γ.

Identification of the potential IL-36 effector cells in the lung is crucial in understanding the role of the elevated levels of IL-36γ in COPD. IL-36 cytokines did not induce chemokine release from TMφ from smokers and COPD subjects. Previous studies have suggested that monocyte-derived M2, but not M1, macrophages release IL-6 in response to IL-36β (46). This is discrepant with the data presented here and may reflect differences between tissue resident cells and monocyte-derived macrophages. Our data are in agreement with others who showed that bronchial epithelial cells stimulated with IL-36γ released CXCL8 and IL-6 (24). In contrast, SAF secreted much higher concentrations of cytokines, chemokines, and active MMPs in response to IL-36 cytokines, suggesting that these are likely to be the major effector cell in the lung. Lung fibroblasts, along with colonic fibroblasts, have previously been shown to secrete cytokines, chemokines, and MMPs in response to IL-36 (20, 47). Epithelial cells grown at air-liquid interface have been shown to release IL-36 cytokines from the basolateral surface; therefore, fibroblasts acting as the main effector cells correlates with this finding (20). IL-36 stimulation also induced extracellular matrix deposition from human fibroblasts (48); however, we found no change in the gene expression of collagen genes and a downregulation of the myofibroblast marker α-SMA in response to IL-36.

Systemic glucocorticosteroids are commonly used in the treatment of COPD exacerbations in an attempt to reduce the increased inflammation associated with these infective episodes. We, therefore, tested whether IL-36γ–mediated inflammation was steroid sensitive. CXCL8, IL-6, and GM-CSF induction by IL-36γ in SAF was steroid sensitive, with release reduced by approximately 50%–60% by high concentrations of budesonide. However, a major neutrophil chemokine, CXCL1, was paradoxically induced by budesonide in the presence of IL-36γ, suggesting that administration of steroids to patients with elevated IL-36γ may be proinflammatory via increased CXCL1 and increased neutrophil recruitment. Previous data have suggested that CXCL1 levels are unaffected by inhaled glucocorticosteroids in patients with COPD, in contrast to CXCL8 (49). In vitro studies are conflicting, with studies suggesting both steroid sensitivity and insensitivity to the same and different stimuli (50, 51). Nevertheless, our data suggest that giving a steroid during an exacerbation when IL-36γ may be induced via a virus could lead to further neutrophil recruitment and, thus, be detrimental to the patient.

SAF release high levels CXCL1 and CXCL8, in response to IL-36γ. These chemokines recruit neutrophils from the blood, and higher numbers of COPD neutrophils migrate to CXCL1 compared with cells from nonsmokers; CXCL1 is markedly elevated in the lungs of patients with COPD (27, 52). To enter the lung, these neutrophils must migrate though the tissue, releasing proteases as they travel. In COPD, this migration is altered with an increase in speed that is less directional (53) and can lead to excess tissue damage. Therefore, the elevation of both CXCL1 and CXCL8 can increase the recruitment of neutrophils from the blood into the COPD lung and, in doing so, can cause excessive tissue damage due to their dysregulated migratory path.

Once in the lung, neutrophils degranulate and release several proteases, including the serine proteases neutrophil elastase, cathepsin G, and proteinase-3. Here, we show that cathepsin G and proteinase-3 cleave IL-36γ into its active form, which has been shown to have 500-fold greater activity than the uncleaved secreted cytokine. These data contrast with others that show that neutrophil elastase is the main activator of IL-36γ (8), but this is also controversial with others failing to show this response (7, 37). However, since neutrophils are increased and activated in COPD, it is plausible that they activate IL-36γ, leading to a marked increases in the potency of IL-36γ within the COPD lung, thus amplifying and perpetuating neutrophilic inflammation.

Finally, we showed that blocking the IL-36R by treating SAF with either recombinant IL-36Ra or an IL-36R blocking antibody reduced CXCL1 release from these cells when stimulated with media from poly(I:C)-stimulated COPD SAEC. These data suggest that reintroducing the natural inhibitor of the IL-36R or directly blocking the receptor with a therapeutic antibody, such as Spesolimab, may reduce viral-induced inflammation via the IL-36 pathway. We have recently shown, in an in vivo model of a COPD viral exacerbations, that KO of IL-36R reduces lung inflammation and neutrophil recruitment (19). Our crosstalk experiments show that attenuating the activity of the IL-36R in primary human cells from COPD subjects may also lead to reduced inflammation, suggesting that this may be translated into humans and may be a potential therapeutic option.

The regulation of IL-36 cytokines is complex, with the requirement for extracellular protease activation and their modulation by the antagonists IL-36Ra and IL-38. These different IL-36 family members appear to be released from different cell types, suggesting a complex interactive cell network (7). The effects of IL-36 cytokines are similar to those of related IL-1 cytokines, which are released predominantly via a different mechanism, involving intracellular activation via the inflammasome (54). It is possible that IL-1 cytokines provide the initial inflammatory process in host defence and that IL-36 cytokines are activated with greater stimulatory triggers or more prolonged stimulation, resulting in greatly amplified and persistent neutrophilic inflammation, as found in patients with COPD. A limitation of the present study is the lack of smoker groups for studies on SAEC and SAF, although the data suggest that, in smokers, IL-36Ra is not downregulated in the lung homogenate samples.

Overall, our data suggest that IL-36γ is elevated in the COPD lung and is released predominantly by epithelial cells, leading to the activation of fibroblasts. This induces neutrophil recruitment, which further activates IL-36γ, inducing protease release and inflammation; all of this activity drives COPD pathophysiology (Figure 10). This mechanism is amplified by a reduction in IL-36Ra from macrophages and by the marked activation of IL-36γ by serine proteases released from the activated recruited neutrophils. We suggest that this is a major mechanism for amplification of lung inflammation in COPD, leading to persistent inflammation and disease progression. This suggests that targeting IL-36 cytokines — for example, with neutralizing antibodies against the receptor or addition of IL-36Ra — is a promising therapeutic opportunity for the treatment of COPD.

## Methods

### Reagents.

Recombinant IL-1α, IL-36α, IL-36β, IL-36γ, IL-36Ra, TNF-α, neutrophil elastase, cathepsin G, proteinase-3, and anti–IL-36γ antibody (catalog AF2320) were purchased from R&D Systems. Rabbit anti–goat Ig/HRP (P0449) was purchased from Agilent. Poly(I:C) was purchased from Sigma-Aldrich. Nontypeable *H*. *influenzae* were obtained from the National Collection of Type Cultures (strain no. 1269) and were heat-killed by incubation at 65°C for 10 minutes as described previously (55). CSE was generated as previously described (56) from full-strength Marlboro cigarette (Phillip Morris). Budesonide was purchased from Thermo Fisher Scientific.

### BAL.

BAL was performed as described previously (31, 57). Briefly, BAL was collected from the right middle lobe by instilling 60 mL of warmed 0.9% (wt/vol) normal saline into the lung to a maximum of 240 mL. BAL was filtered and centrifuged (500*g* for 10 minutes at 4°C) to remove cells and stored at –80°C. See Supplemental Table 1 for patient demographics.

### Induced sputum.

Induced sputum was collected and processed following a modification of Pin et al. (58) as reported previously (59). Briefly, subjects inhaled increasing concentrations of hypertonic saline solution (3%, 4%, and 5% [wt/vol]) for 7 minutes at each concentration. The opaque, gelatinous portions of sputum were selected and centrifuged (300*g* for 10 minutes at 4°C). The viscous sample was weighed and treated dithiothreitol (DTT) diluted to 0.1% (wt/vol) with distilled water. The sample was solubilized by vortexing. Four volumes of Dulbecco PBS solution were added to the sample to give a final concentration of 0.05% (wt/vol) DTT. The supernatant was collected and stored at −80°C (59). See Supplemental Table 2 for patient demographics.

### Nasosorption.

Nasosorption was performed using Nasosorption FX·I device (Hunt Developments UK Ltd.) as described by others (60–63). Briefly, a Nasosorption FX·I device containing a synthetic absorptive matrix (SAM) was inserted into each nostril for 60 seconds for sample collection. Each SAM was detached and placed into 300 μL of elution buffer, vortexed for 30 seconds, and transferred to a Costar SPIN-X bucket (MilliporeSigma). The sample was then centrifuged for 20 minutes at 16,000*g* in a minicentrifuge cooled to 4°C. Supernatant was then stored at −80°C. See Supplemental Table 3 for patient demographics.

### Primary human lung cells.

Lung tissue macrophages were isolated from lung parenchyma tissue, as described previously (64, 65). Lung tissue was assessed as being noncancerous and obtained from samples during tissue resection for lung cancer or emphysema. The subjects were matched for age (Supplemental Table 4) Human primary SAECs were cultured as previously described (66). The subjects were matched for age (Supplemental Table 5). SAFs were cultured by microdissecting out 5 small airways from lung parenchymal tissue and by growing them via an outgrowth method (Supplemental Table 6). Lung homogenate samples were obtained from an established tissue bank linked to an established patient registry, which has previously been used (67) (Supplemental Table 7). COPD subjects had significantly worse lung function compared with controls.

### Real-time PCR.

Total RNA was extracted from cells and reverse-transcribed, as described previously (66). Gene expression was determined by Taqman real-time PCR on a 7500 Real Time PCR system (Applied Biosystems) using the assays IL-36α (Hs00205367), IL-36β (Hs00758166), IL-36γ (Hs00219742), IL-36Ra (Hs01104220), CXCL8 (Hs00174103), IL-6 (Hs00174131), COL1A1 (Hs00164004), COL3A1 (Hs00943809), α-SMA (Hs05032285), and MMP9 (Hs00957562). GNB2L1 (Hs00272002) gene expression was used as the housekeeping gene, and data are presented as ΔΔCT relative to baseline.

### Zymography.

MMP2 and MMP9 enzyme activity were measured by zymography using Novex Zymogram Gelatin Gels (Thermo Fisher Scientific). Fibroblast supernatant were diluted in Novex Tris-Glycine SDS sample buffer (Thermo Fisher Scientific) and were run on zymogram gels. After electrophoresis, gels were incubated with Novex zymogram renaturing buffer (Thermo Fisher Scientific) and incubated in Novex zymogram developing buffer (Thermo Fisher Scientific) for 18 hours at 37°C. After incubation, gels were stained with a Colloidal Blue Staining Kit (Thermo Fisher Scientific) and imaged.

### ELISA.

CXCL8, CXCL1, IL-6, GM-CSF, IL-36α, and IL-36β were quantified using commercially available ELISA kits (R&D Systems), according to the manufacturer’s instructions. The lower limits of detection for these assays were 31.2 pg/mL (CXCL1/8, IL-6), 15.6 pg/mL (GM-CSF), and 12.5 pg/mL (IL-36α/β). IL-36γ and IL-36Ra were quantified using commercially available ELISA kits (AdipoGen life sciences). The lower limits of detection for these assays were 3.9 pg/mL (IL-36γ) and 0.5 ng/mL (IL-36Ra).

### IL-36γ cleavage.

Neutrophils were isolated from whole blood from both nonsmokers and COPD subjects using dextran RBC sedimentation, followed by centrifugation (750*g* at room temperature) using a discontinuous Percoll gradient. Neutrophils were collected from the 81% (v/v)/67% (v/v) interface and washed in PBS; cells were resuspended at 1 × 10^6^ cells/mL in Reaction Buffer (50 mM HEPES [pH 7.5], 75 mM NaCl, 0.1% [w/v] CHAPS). Cells were treated with 10 μM cytochalasin prior to stimulation with 10 μM fMLP for 1 hour (or 0.1% [v/v] DMSO vehicle control) at 37°C. Cells were then centrifuged at 500*g* at room temperature, and the supernatant was removed and stored at –80°C. IL-36γ (uncleaved-1F9) at 1000 ng/mL was then incubated at 37°C for 2.5 hours with nonsmoker and COPD neutrophil supernatants, cathepsin G (10, 100, and 1000 nM) (MilliporeSigma), proteinase-3 (2, 10, and 50 μg/mL) (MilliporeSigma), neutrophil elastase (10, 100, and 1000 ng/mL) (MilliporeSigma), or MMP9 at (1, 10, and 100 ng/mL) (R&D Systems Europe) in protease buffer (50 mM HEPES [pH 7.5], 75 mM NaCl, 0.1% CHAPS). In total, 10 μL (100 ng) of reaction buffer was removed and boiled with Lamaelli buffer (Thermo Fisher Scientific) for 5 minutes before loading onto a 4%–12% gel using MES buffer (Thermo Fisher Scientific). Proteins were transferred to a nitrocellulose membrane before probing using a primary anti IL-36γ antibody (R&D Systems) overnight at 4°C and a secondary goat antibody (Dako) for 1 hour at room temperature to visualize any protein cleavage.

### IL-36Ra and IL-36R antibody treatment experiments.

SAEC from 3 COPD subjects were treated with poly(I:C) (100 μg/mL) for 24 hours to induce IL-36γ, and supernatants were collected and pooled. For IL-36Ra experiments, pooled supernatant was transferred to COPD SAF and incubated for 24 hours; controls of media alone and/or media containing poly(I:C) (100 μg/mL) were used. SAF cells were then treated for 2 hours with 100 ng/mL of recombinant IL-36Ra, before being treated with SAEC media. For IL-36R antibody experiments, SAF were pretreated for 2 hours with either IgG1 isotype control or human anti–IL-36R Ab (Boehringer Ingelheim) at 50 μg/mL in sterile PBS, before being treated with SAEC media. In these experiments, a maximum of 340 pg/mL of CXCL1 could have been transferred in the SAEC media to the SAFs; therefore, release above this level was because of mediators within the media. To show that the induction to CXCL1 in the SAF was a consequence of a mediator within the media, the media was boiled for 5 minutes at 100°C, and SAF treated with this media and CXCL1 release measured.

### Statistics.

Data are expressed as means ± SEM. Results were analyzed by using Mann-Whitney *U* tests, paired or nonpaired 2-tailed Student’s *t* tests, 1- or 2-way ANOVA, and Kruskal-Wallis for repeated measures with Dunn’s or Bonferroni post hoc tests. GraphPad Prism 9 software (GraphPad Software) was used for analyzes. *P* ≤ 0.05 was considered statistically significant.

### Study approval.

For BAL and sputum samples, subjects provided written informed consent, and the study was approved by NRES London-Chelsea Research Ethics committee (study 09/H0801/85). For the collection of human tissue, subjects provided written informed consent and the study was approved by NRES South Central-Hampshire B Research Ethics committee 15/SC/0101. The samples are taken from nonoverlapping subjects and from different cohorts of patients.

## Author contributions

JRB was involved in the design, implementation of the experiments, and the writing of the manuscript. PSF and HBO were involved in the implementation of the experiments, and they provided technical expertise. CKK, KCEK, MT, JF, SLE, PJB, and LED were involved in experimental design, interpretation of data, and the review of the manuscript. All authors contributed to scientific discussions and revision of the manuscript, and they had full access to all the data and agreed to submit for publication.

## Supplementary Material

Supplemental data

## Version 1 06/28/2022

In-Press Preview

## Version 2 08/08/2022

Electronic publication
