# Supplementary material for: IL-36 receptor agonist and antagonist imbalance drives neutrophilic inflammation in COPD
Source: JCI Insight. 2022 Aug 8;7(15):e155581. doi: 10.1172/jci.insight.155581 (PMC9462491; doi:10.1172/jci.insight.155581)
Supplement: Supplemental data [file jciinsight-7-155581-s096.pdf]

Supplementary figures

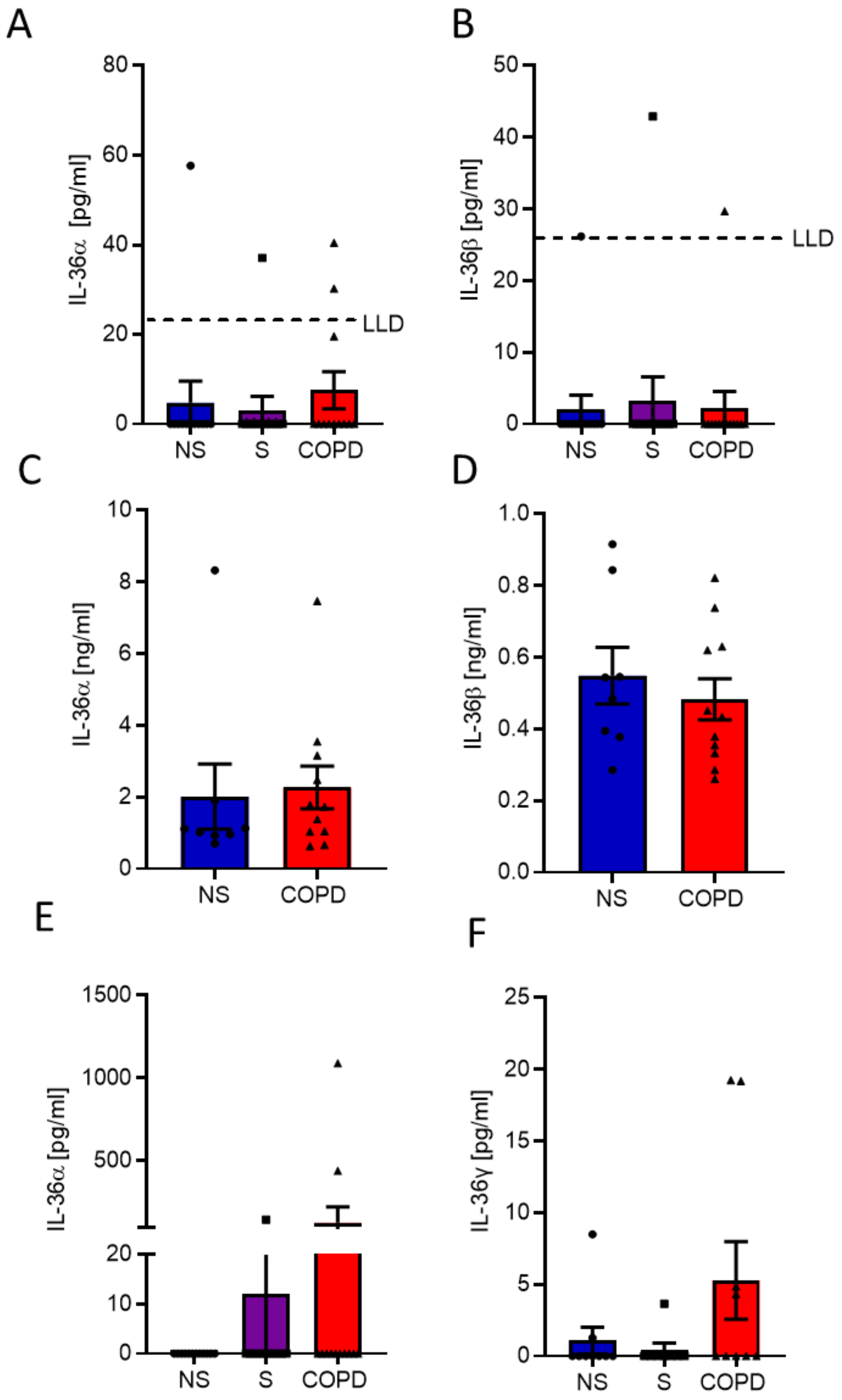

Supplementary Figure 1. IL-36 cytokines levels in human samples.
A) IL-36α and B) IL-36β were measured in bronchoalveolar lavage fluid of non-smokers (n=12), smokers (n=11) and COPD (n=11) patients by ELISA. C) IL-36α and D) IL-36β were measured in nasal lining fluid of non-smoker (n=8) and COPD (n=11) patients by ELISA. E) IL-36α and F) IL-36γ were measured in serum samples of non-smokers (n=11) smokers (n=12) and COPD (n=12) patients by ELISA. Data are mean ± SEM and LLD = lower limit of detection

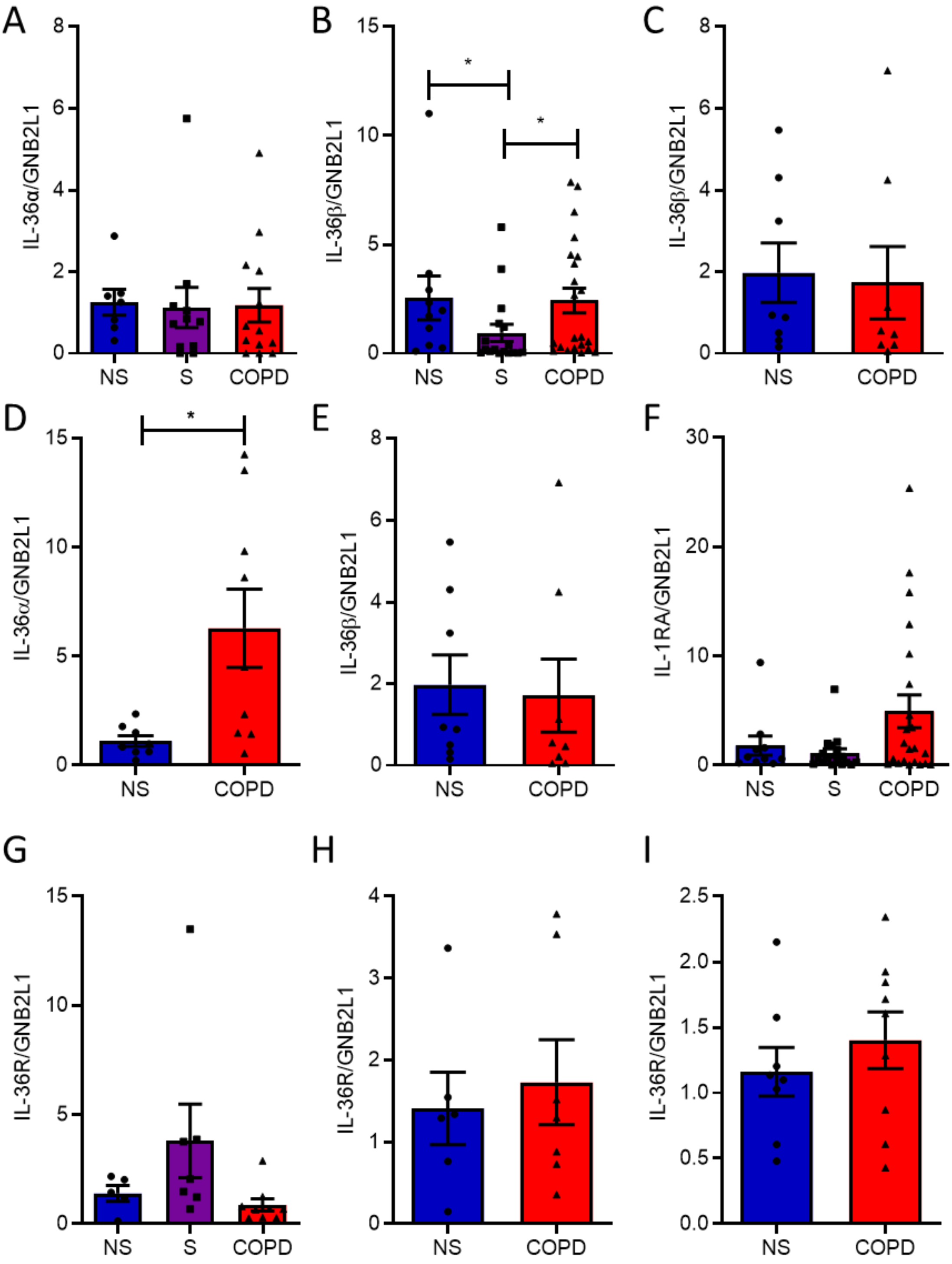

A
B
C
D
E
F
G
H
I
IL-36α/GNB2L1
IL-36β/GNB2L1
IL-1RA/GNB2L1
IL-36R/GNB2L1
NS
S
COPD
*

Supplementary Figure 2. Expression of IL-36 isoforms and receptor in human lung tissue macrophages, small airway fibroblasts and small airway epithelial cells. A) *IL-36A* and B) *IL-36B* gene expression were measured in lung tissue macrophages from non-smokers (NS) (n=7-11), smokers (S) (n=11-17) and COPD patients (n=13-22). C) IL-36β gene expression was measured in SAF from non-smokers (n=8) and COPD patients (n=8). Gene expression of D) IL-36A and E) IL-36B were measured in small airway epithelial cells from non-smokers (n=8) and COPD patients (n=9). F) IL-1RA gene expression was detected in non-smoker (n=10), smoker (n=17) and COPD (n=22) tissue macrophages. IL-36 receptor gene expression was measured in G) tissue macrophages H) SAEC and I) SAF. Data are means ± SEM and analyzed by Kruskal-Wallis test with post-hoc Dunn's test or by Mann-Whitney U test; * $P < 0.05$.

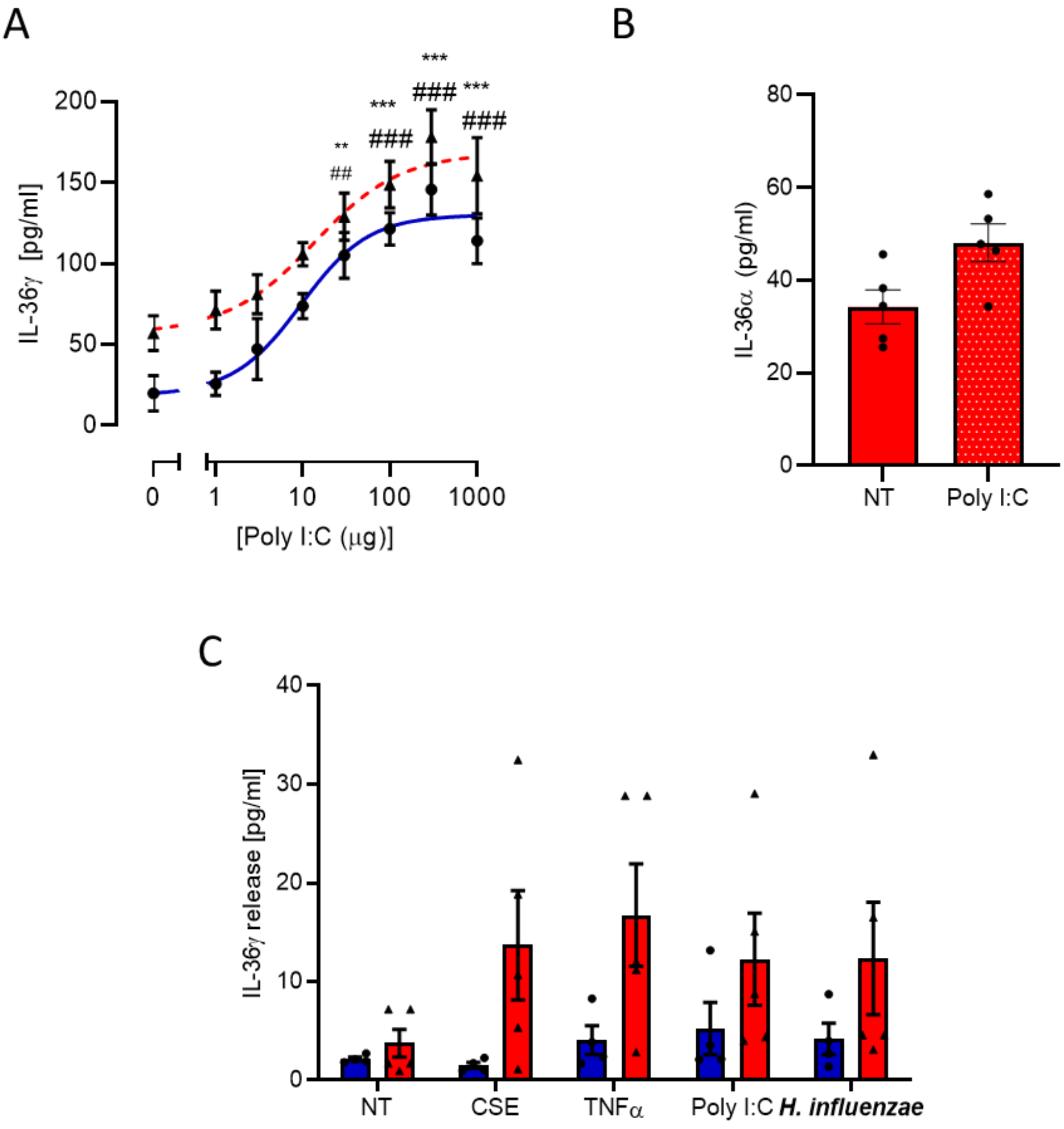

Supplementary Figure 3. Poly I:C induces IL-36γ release from SAEC, but not SAF, whilst not inducing IL-36α. A) Small airway epithelial cells from non-smokers (NS, ●, blue n=3) and COPD patients (▲, red n=3) were exposed to increasing concentrations of poly I:C for 24h. Media was collected and IL-36γ was measured by ELISA. B) SAEC were treated with 100 μg/ml of Poly I:C for 24 hours and IL-36α release detected by ELISA. C) SAF from non-smokers (NS, ●, blue, n=4) and COPD patients (▲, red, n=5) were exposed to media alone (no treatment: NT), 10% (v/v) cigarette smoke extract (CSE), 10 ng/ml TNFα, 100μg/ml poly I:C, or 1.5×10^{10} CFU/ml *H. influenzae* for 24h. Media was collected and IL-36γ release measured by ELISA. Data are means ± SEM and analyzed by Kruskal-Wallis test with post-hoc Dunn's test ** $P < 0.01$ and *** $P < 0.001$ for COPD subjects and $^{\#\#}$ $P < 0.01$ and $^{\#\#\#}$ $P < 0.001$ for NS.

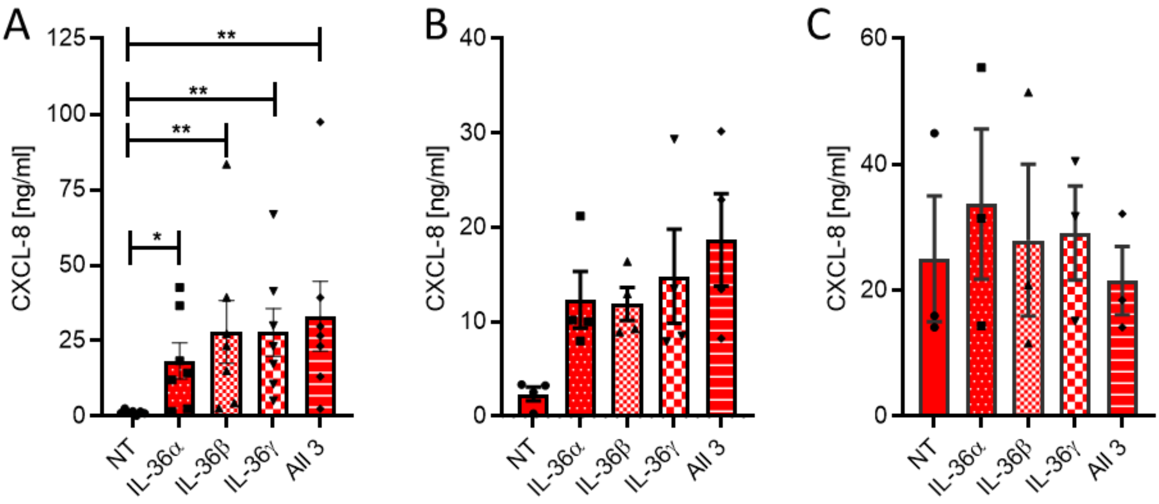

Supplementary Figure 4. Effect of IL-36 cytokines alone and in combination on CXCL-8 release from small airway fibroblasts, human small airway epithelial cells and lung tissue macrophages. A) Small airway fibroblasts, B) small airway epithelial cells and C) lung tissue macrophages were incubated in the absence (non-treated: NT) or presence of 33 ng/ml IL-36α, IL-36β, IL-36γ or all three in combination for 24h. Media was harvested, and release of CXCL-8 measured by ELISA. Data are means ± SEM and analyzed by Kruskal-Wallis test with post-hoc Dunn's test; * P <0.05, ** P<0.01.

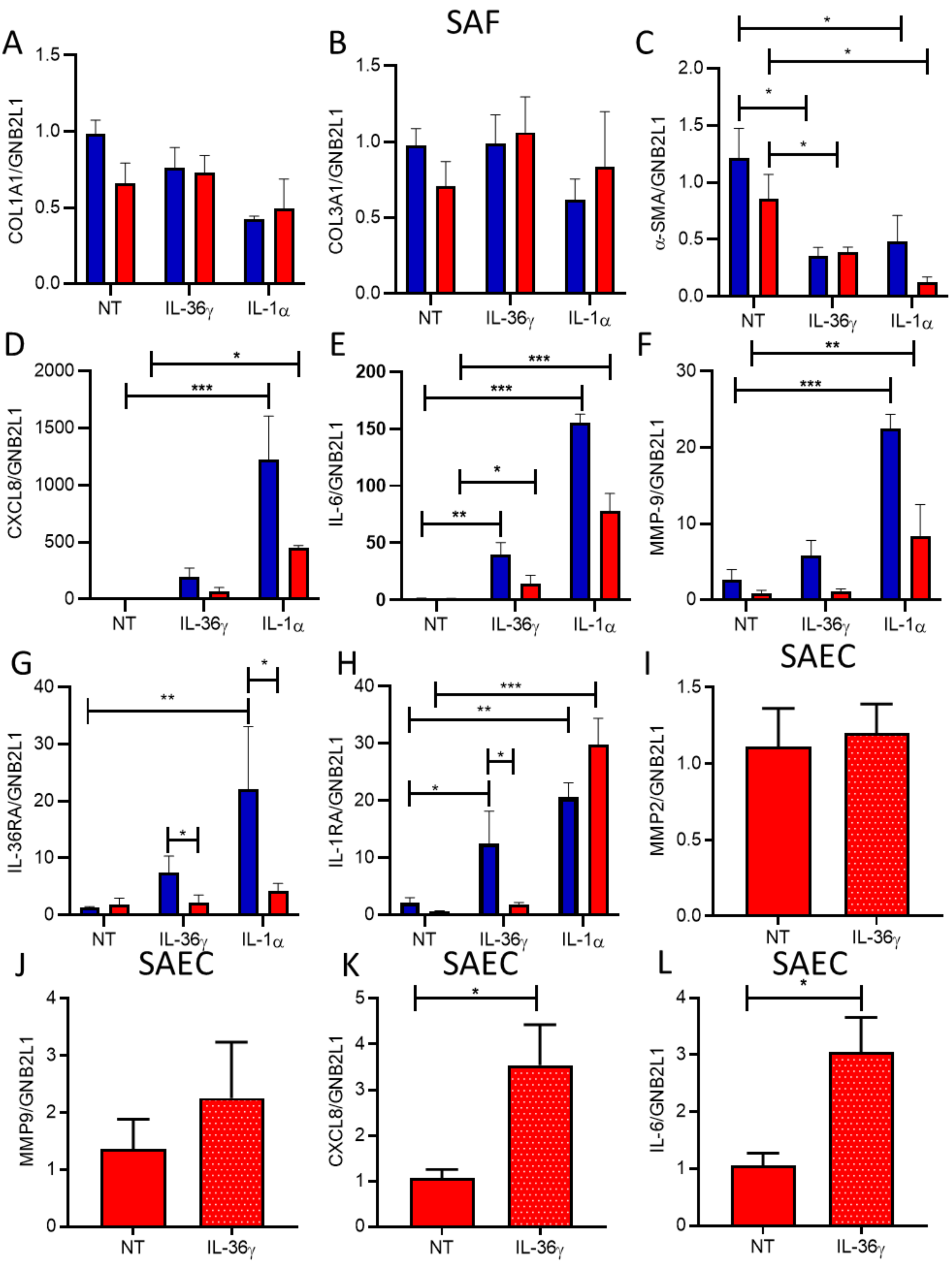
SAF
A
COL1A1/GNB2L1
B
COL3A1/GNB2L1
C
α-SMA/GNB2L1
D
CXCL8/GNB2L1
E
IL-6/GNB2L1
F
MMP-9/GNB2L1
G
IL-36RA/GNB2L1
H
IL-1RA/GNB2L1
I
SAEC
MMP2/GNB2L1
J
SAEC
MMP9/GNB2L1
K
SAEC
CXCL8/GNB2L1
L
SAEC
IL-6/GNB2L1
NT
IL-36γ
IL-1α

Supplementary Figure 5. Effect of IL-36γ and IL-1α cytokines on gene expression in small airway fibroblasts (SAF) and small airway epithelial cells (SAEC). Small airway fibroblasts from non-smokers (n=7, blue) or COPD (n=7, red) were treated with 100 ng/ml of IL-36γ or IL-1α ng/ml for 24h. RNA was collected and gene expression of A) COL1A1, B) COL3A1, C) α-SMA and D) CXCL8, E) IL-6, F) MMP-9, G) IL-36RN and H) IL-1RA were detected by qRT-PCR. Small airway epithelial cells (n=4) were treated with 100 ng/ml of IL-36γ for 24h. RNA collected and gene expression of I) MMP-2, J) MMP-9, K) CXCL8, and L) IL-6, were detected by qRT-PCR Data are means ± SEM and analyzed by two-way anova with post-hoc Dunnett's multiple comparisons test (panel A-H) or by Mann-Whitney U test (panel I-L) ; * P <0.05, ** P<0.01, *** P<0.001.

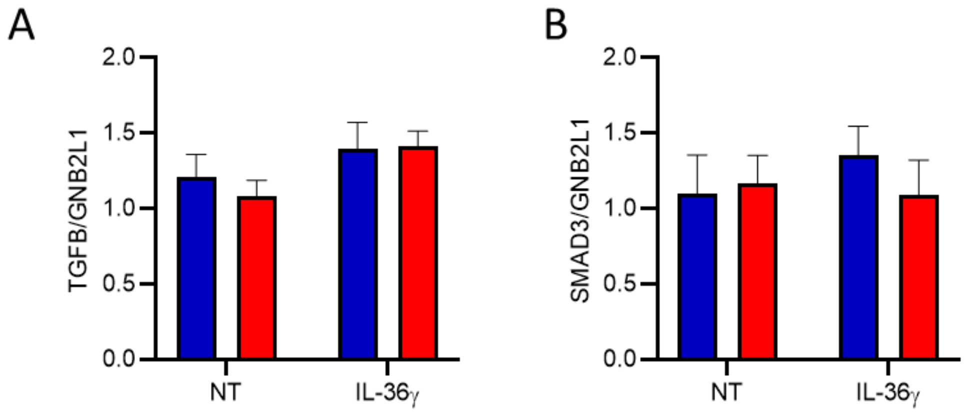

Supplementary Figure 6. Effect of IL-36γ on gene expression in small airway fibroblasts (SAF). Small airway fibroblasts from non-smokers (n=6, blue) or COPD (n=6, red) were treated with 100 ng/ml of IL-36γ for 24h. RNA was collected and gene expression of A) TGFBβ, and B) SMAD3 were detected by qRT-PCR. Data are means ± SEM.

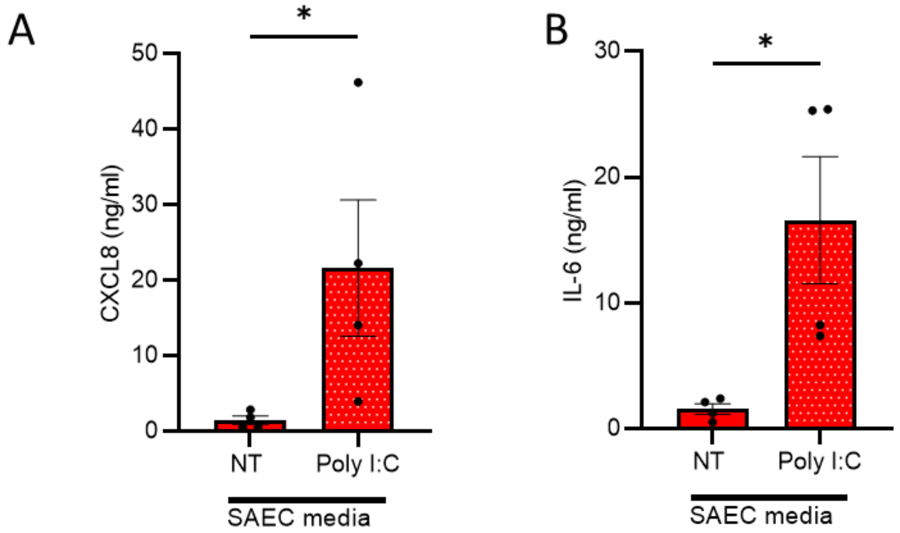

Supplementary Figure 7. Poly I:C stimulated SAEC conditions media induces release of other cytokines from SAF. Small airway fibroblast (n=4) were treated with diluted (ranging from 10-200 fold) media from untreated or Poly I:C treated SAEC and A) CXCL8 and B) IL-6 release were detected by ELISA. Data are means ± SEM and analyzed by Mann-Whitney U test; * P <0.05.

Supplementary Table 1. The characteristics of subjects for bronchoscopy

	Non-smoker	Smoker	COPD
Sex (F:M)	2:8	3:7	5:5
Age (years)	62±10.6	56±6.2	64.5±13.2
Smoking history (pack-years)	0	27.8±10	37.8±14.6
FEV_1 (L)	3.2±0.5	2.9±0.7	1.6±0.5*
FEV_1 (% predicted normal)	100.2±10.0	86.6±15.9	62.9±22.7**
FVC (L)	3.9±0.75	3.9±1.0	2.9±0.75
FEV_1/FVC	0.79±0.1	0.75±0.1	0.52±0.1***

Abbreviations: COPD = chronic obstructive pulmonary disease; FEV_1 = forced expiratory volume in one second; FVC = forced vital capacity; Data are expressed as mean value ± standard deviation and by Kruskal-Wallis test with post-hoc Dunn's test; * P <0.05, **P<0.01 and *** P<0.001 compared to non-smokers.

Supplementary Table 2. The characteristics of study subjects for sputum samples

	Non-smoker	Smoker	COPD
**Sex (F:M)**	9:9	4:4	7:13
**Age (years)**	46.25±11.05	55.8±3.2	68.6±9.7**
**Smoking history (Pack-years)**	0	37±8.6***	44.7±31.6***
**FEV_1 (L)**	2.9±0.9	2.67±0.5	1.46±0.7***
**FEV_1 (% predicted normal)**	97.4±17.3	95.8±13.3	55.6.1±26.5***
**FVC (L)**	3.7±1.2	2.6±0.5	3.1±0.9
**FEV_1/FVC**	0.78±0.7	0.77±0.1	0.46±0.2***

Abbreviations: COPD = chronic obstructive pulmonary disease; FEV_1 = forced expiratory volume in one second; FVC = forced vital capacity. Data are expressed as mean value ± standard deviation and by Kruskal-Wallis test with post-hoc Dunn’s test; **P<0.01 and *** P<0.001 compared to non-smokers.

Supplementary Table 3. The characteristics of study subjects for nasal absorption samples

	Non-smoker	COPD
**Sex ratio (F:M)**	6:2	8:12
**Age (years)**	61.1±3.2	66.5±2.1
**Smoking History (pack years)**	0.0±0.0	51.47±7.5***
**FEV_1 (L)**	2.8±0.3	1.4±0.6**
**FEV_1 % Predicted**	109.4±5.5	54.75±4.3***
**FVC (L)**	3.8±0.5	3.0±1.9
**FEV_1:FVC**	0.75±0.0	0.48±0.0***

Abbreviations: COPD = chronic obstructive pulmonary disease; FEV_1 = forced expiratory volume in one second; FVC = forced vital capacity. Data are expressed as mean value ± standard deviation and analyzed by Mann-Whitney U test; * P <0.05 ***P<0.001 compared to non-smokers.

Supplementary Table 4. The characteristics of study subjects for primary tissue macrophages

	Non-smoker	Smoker	COPD
**Sex (F:M)**	7:3	10:14	13:16
**Age (years)**	69±7.7	66±12.2	69±8.9
**Smoking history (Pack-years)**	0	35.1±27.8***	38.6±18.1**
**FEV_1 (L)**	2.2±0.7	2.5±0.6	1.2±0.6*
**FEV_1 (% predicted normal)**	92.2±24.4	103.2±22.5	52.1.1±29.6**
**FVC (L)**	2.6±0.8	3.5±0.8	2.7±1.1
**FEV_1/FVC**	0.8±0.1	0.72±0.1	0.43±0.1***

Abbreviations: COPD = chronic obstructive pulmonary disease; FEV_1 = forced expiratory volume in one second; FVC = forced vital capacity. Data are expressed as mean value ± standard deviation and by Kruskal-Wallis test with post-hoc Dunn's test; * P <0.05, **P<0.01 and *** P<0.001 compared to non-smokers.

Supplementary Table 5. The characteristics of study subjects for primary airway epithelial cells

	Non-smoker	COPD
**Sex ratio (F:M)**	7:4	10:7
**Age (years)**	71.7±4.3	68.9±6.5
**Smoking History (pack years)**	0.0±0.0	41.3±13.2**
**FEV_1 (L)**	2.1±0.6	1.1±0.89*
**FEV_1 % Predicted**	95.5±19.3	50.6±25.8**
**FVC (L)**	2.8±0.7	2.5±0.8
**FEV_1:FVC**	0.74±0.1	0.5±0.2**

Abbreviations: COPD = chronic obstructive pulmonary disease; FEV_1 = forced expiratory volume in one second; FVC = forced vital capacity; Data are expressed as mean value ± standard deviation and analyzed by Mann-Whitney U test; * $P<0.05$ **$P<0.01$ compared to non-smokers.

Supplementary Table 6. The characteristics of study subjects for primary small airway fibroblasts

	Non-smoker	COPD
**Sex ratio (F:M)**	7:9	7:5
**Age (years)**	64.6±9.1	65.5±8.7
**Smoking History (pack years)**	0.0±0.0	34.5±18.3**
**FEV_1 (L)**	2.7±0.9	1.5±0.8*
**FEV_1 % Predicted**	92.7±11.6	52.9±25.6***
**FVC (L)**	3.4±0.8	3.2±1.1
**FEV_1:FVC**	0.79±0.1	0.54±0.2**

Abbreviations: COPD = chronic obstructive pulmonary disease; FEV_1 = forced expiratory volume in one second; FVC = forced vital capacity; Data are expressed as mean value ± standard deviation and analyzed by Mann-Whitney U test; * P <0.05, **P<0.01 and *** P<0.001 compared to non-smokers

Supplementary Table 7. The characteristics of study subjects for lung homogenate samples

	**Non-smokers (n=9)**	**Smokers (n=9)**	**GOLD Stage 1 (n=9)**	**GOLD Stage 2 (n=9)**	**GOLD Stage 3 (n=3)**	**GOLD Stage 4 (n=6)**
**Age** (years)	63.4±13.6	63±12.3	67.7±7.0	63.0±9.3	62.3±10.9	59.8±4.5
**Sex** (F:M)	7:2	5:4	3:5	5:4	1:2	4:2
**FEV_1** (L)	2.56±0.6	2.8 ±0.6	2.7±0.6	1.8±0.4*	1.69±0.4*	0.5±0.18**
**FEV_1** (% predicted)	97.2±16.4	99.4±13.3	89.1±3.9	65.4±17.5**	49.7±4.3*	16.1±2.9***
**FVC** (L)	3.2±1.1	3.6±0.9	4.0±0.9	3.1±0.8	3.0.1±0.7	1.75±0.5*
**FEV_1:FVC**	80.3±4.9	75.4±4.1	64.3±3.6*	61.5±7.9**	51.8±7.1*	28.5±8.1***
**Smoking History** (Pack years)	0±0	61.1±32.4	44.3±17.0	57.7±35.4	46.6±21.8	38.6±15.9

Abbreviations: COPD = chronic obstructive pulmonary disease; FEV_1 = forced expiratory volume in one second; FVC = forced vital capacity. Data are expressed as mean value ± standard deviation and by Kruskal-Wallis test with post-hoc Dunn's test; * P <0.05, **P<0.01 and *** P<0.001 compared to non-smokers.
